# A Circular RNA Expressed from the *FAT3* Locus Regulates Neural Development

**DOI:** 10.1007/s12035-023-03253-7

**Published:** 2023-02-25

**Authors:** Sabine Seeler, Maria Schertz Andersen, Tamas Sztanka-Toth, Mateja Rybiczka-Tešulov, Marleen H. van den Munkhof, Chi-Chih Chang, Muyesier Maimaitili, Morten Trillingsgaard Venø, Thomas Birkballe Hansen, R. Jeroen Pasterkamp, Agnieszka Rybak-Wolf, Mark Denham, Nikolaus Rajewsky, Lasse Sommer Kristensen, Jørgen Kjems

**Affiliations:** 1 Interdisciplinary Nanoscience Center, Department of Molecular Biology and Genetics, Aarhus University, 8000 Aarhus C, Aarhus, Denmark; 2 Department of Biomedicine, The Skou Building, Aarhus University, 8000 Aarhus C, Aarhus, Denmark; 3 Berlin Institute for Medical Systems Biology (BIMSB), MDC Berlin-Mitte, 10115 Berlin, Germany; 4 Department of Translational Neuroscience, University Medical Center Utrecht Brain Center, 3584 CG, Utrecht, Netherlands; 5 Danish Research Institute of Translational Neuroscience, Nordic EMBL Partnership for Molecular Medicine, Aarhus University, 8000 Aarhus C, Aarhus, Denmark; 6 Omiics ApS, 8200 Aarhus N, Aarhus, Denmark

**Keywords:** ncRNAs, circRNAs, Cerebral organoids, scRNA-seq, Neural development, circFAT3

## Abstract

Circular RNAs (circRNAs) are key regulators of cellular processes, are abundant in the nervous system, and have putative regulatory roles during neural differentiation. However, the knowledge about circRNA functions in brain development is limited. Here, using RNA-sequencing, we show that circRNA levels increased substantially over the course of differentiation of human embryonic stem cells into rostral and caudal neural progenitor cells (NPCs), including three of the most abundant circRNAs, ciRS-7, circRMST, and circFAT3. Knockdown of circFAT3 during early neural differentiation resulted in minor transcriptional alterations in bulk RNA analysis. However, single-cell transcriptomics of 30 and 90 days differentiated cerebral organoids deficient in circFAT3 showed a loss of telencephalic radial glial cells and mature cortical neurons, respectively. Furthermore, non-telencephalic NPCs in cerebral organoids showed changes in the expression of genes involved in neural differentiation and migration, including *FAT4*, *ERBB4*, *UNC5C*, and *DCC*. In vivo depletion of circFat3 in mouse prefrontal cortex using in utero electroporation led to alterations in the positioning of the electroporated cells within the neocortex. Overall, these findings suggest a conserved role for circFAT3 in neural development involving the formation of anterior cell types, neuronal differentiation, or migration.

**Supplementary Information:**

The online version contains supplementary material available at 10.1007/s12035-023-03253-7.

## Introduction

The mammalian nervous system develops through a succession of tightly orchestrated processes including neural induction, neural tube patterning along the anterior–posterior and dorsal–ventral axes, progenitor cell expansion, neural differentiation, and neuronal migration [1, 2]. Several types of non-coding RNAs (ncRNAs), including microRNAs (miRNAs) [3] and long ncRNAs (lncRNAs) [4, 5], have been described to fine-tune the gene expression patterns leading to a fully functional nervous system. Accordingly, circRNAs have been hypothesized to be yet another class of ncRNAs with gene-regulatory potential in neural development [6–13].

During the last decade, circRNAs have become recognized as important regulators of cellular processes (reviewed in [14]) with diverse mechanisms of action, including miRNA sponging [8, 9, 15, 16], transcriptional regulation [17], and interaction with RNA-binding proteins [18–22], and some circRNAs may also function as templates for peptide production [23–26].

CircRNAs are expressed in a wide range of organisms from archaea to mammals [10, 27–30] and many are evolutionarily conserved and expressed in a cell type- and a developmental stage-specific manner [8–10, 28, 31]. This is particularly striking in the nervous system, where circRNAs are highly abundant relative to other tissues [8, 11, 12, 30, 32]. Furthermore, circRNA expression has been shown to increase during neural differentiation [10–12, 33, 34]. CircRNAs are predominantly localized in the cytosol but may also reside in the nucleus or within membrane-less subcellular compartments, like synaptoneurosomes. This points towards a possible function in synapse formation or transmission in the developing nervous system [11, 12]. However, only few individual circRNAs have been found to influence the development of the mammalian nervous system. CircSLC45A4 induces spontaneous neuronal differentiation in the human neuroblastoma cell line, SH-SY5Y, in vitro and alters basal progenitor and Cajal-Retzius cell numbers in murine cortical development in vivo [13], whereas circZNF827 regulates neuronal gene expression (e.g., *NGFR* and *RARA*) upon interaction with the inhibitory complex hnRNP K/L in the nucleus [7]. In addition, the overexpression of murine ciRS-7 (also known as CDR1as) resulted in midbrain defects in embryonic zebrafish CNS, which may have been potentiated by downregulating miR-7 [8]. In contrast, ciRS-7 KO mice showed downregulation of miR-7 and upregulation of miR-7 target genes as well as impaired synaptic transmission and subtle changes in cognitive behavior [6].

In the current study, we aimed to identify circRNAs that potentially regulate neural development. Initially, we used human 2D in vitro models to map global circRNA expression patterns during early neural development. We selected three of the most abundant and upregulated circRNAs, ciRS-7, circRMST, and circFAT3, for subsequent loss-of-function analyses in the 2D model. Based on these analyses, circFAT3 was selected for further knockdown studies in a human 3D cerebral organoid (CO) model. Bulk and single-cell RNA-sequencing (RNA-seq and scRNA-seq, respectively) was conducted to assess cell type-specific transcriptomic changes after 30 and 90 days of differentiation. Finally, we used in utero electroporation (IUE) of murine embryonic prefrontal cortex to study the in vivo implications of circFat3 depletion in the developing neocortex.

## Materials and Methods

### Animals

All animal work has been performed in accordance with institutional guidelines, the Dutch law (Wet op Dierproeven 1996), European regulations (Guideline 86/609/EEC), and in agreement with the Animal welfare body. Embryonic day 0 (E0) was counted as the day on which the vaginal plug was detected. C57BL/6 (Charles River) mice were used for all IUE experiments.

### Cell Culture

H9 human embryonic stem cells (hESCs) (WA-09, WiCell) were maintained on feeder-free vitronectin-coated (STEMCELL Technologies, BC, Canada) 35-mm plates in TeSR-E8 medium (STEMCELL Technologies) supplemented with 1% penicillin/streptomycin (p/s; Thermo Fisher Scientific, MA, USA). HEK293T cells were maintained in high glucose Dulbecco’s modified Eagle’s media (DMEM) (Thermo Fisher Scientific) with 10% fetal bovine serum (FBS; Gibco-Thermo Fisher Scientific) and 1% p/s. SH-SY5Y cells were maintained in DMEM/F12 medium (Thermo Fisher Scientific) with 10% FBS and 1% p/s. SH-SY5Y cell differentiation was performed as described previously [12]. P19 cells were maintained in DMEM, high glucose, GlutaMAX medium (Thermo Fisher Scientific) supplemented with MEM non-essential amino acids (NEAA; Thermo Fisher Scientific), 10% FBS, and 1% p/s. All cells were maintained at 37 °C and 5% CO_2_.

### Neural Differentiation of H9 hESCs: 11-Day Differentiation into Rostral and Caudal NPCs

H9 hESCs were transferred to vitronectin-coated 4-well plates in N2B27 medium (1:1 mix of neurobasal medium and DMEM/F12 medium, 1% insulin/transferrin/selenium, 1 × N2 supplement, 1 × retinol-free B27 supplement, 0.3% glucose, and 1% p/s (Thermo Fisher Scientific)) for 11 days of differentiation. As previously described [35], for rostral neural progenitor cell (NPC) differentiation, SB431542 (SB, 10 µM; Tocris, Bristol, UK) and LDN193189 (LDN, 0.1 µM; STEMCELL Technologies) were added to the media for 4 days. The cells were further cultured with SB, LDN, and FGF2 (20 ng/ml; STEMCELL Technologies) until day seven. Between day 7 and 11, the cell media was only supplemented with FGF2 (20 ng/ml). For caudal NPC differentiation, SB431542 (10 µM) and CHIR99021 (CHIR; 3 µM; STEMCELL Technologies) were added to the media from days zero to four. The cells were further cultured from days 4 to 11 in the absence of SB and CHIR, but with FGF2 (20 ng/ml). RNA was harvested at days 0 (before differentiation), 4, and 11.

### Neural Differentiation of H9 hESCs: Cerebral Organoids

H9 hESC-derived cerebral organoids (COs) were generated as described previously [36] with modifications [37, 38]. For a detailed method, see Online Resource 1.

### Plasmids

The empty AGO-dependent short hairpin RNA (agoshRNA) expression vector was modified from the pKHH030 sgRNA vector (Addgene #89,358) to obtain an agoshRNA expression vector with an adenosine (A) at the transcriptional start site of the U6 promoter, an A-C mismatch below the stem of the agoshRNA, and a transcriptional stop site immediately following the agoshRNA. In brief, PCR amplification from the pKHH030 vector was performed using Phusion Hot start flex (New England Biolabs, NEB) with the following primers: 5′-GGGACAGCAGAGATCCAGTT-3′ (FW) and 5′-TAATGGATCCGAAAAAAAGAGGTCTTCTCGAAGACACGGTGTTTCGTCCTTTCCACA-3′ (RE), followed by a restriction enzyme digest using FastDigest NotI and BamHI (Thermo Fisher Scientific). The digested product was then ligated into the original vector using T4 DNA ligase (Thermo Fisher Scientific). Single-stranded DNA oligos encoding agoshRNAs (Sigma) targeting the back splicing junction (BSJ) of circRMST (circBase ID: hsa_circ_0099634), circFAT3 (circBase ID: hsa_circ_0000348), and ciRS-7 (circBase ID: hsa_circ_0001946) were ordered with BbsI overhangs (see Table S1). The DNA oligos were annealed in ligase buffer, phosphorylated using T4 PNK (Thermo Fisher Scientific), and ligated into the BbsI digested empty vector using T4 DNA ligase.

To specifically knockdown mouse circFat3 (circBase ID: mmu_circ_0001746), two BSJ-targeting sequences were designed (Table S1). The first sequence #1 was placed 3 nt upstream, and sequence #2 was placed 3 nt downstream of the BSJ. The biosettia loop (https://biosettia.com/support/shrna-designer/seqreview/) was placed in between two 21 nt long BSJ-targeting sequences including an Xho1 site and a termination signal. pLentiLox3.7 (pll3.7) [39] was used as a backbone and was a gift from Luk Parijs (Addgene plasmid #11,795; http://n2t.net/addgene:11795; RRID:Addgene_11795). For cloning details, see Online Resource 1.

### Transfection of agoshRNA-Constructs and shRNA-Constructs

For transfections of HEK293T and SH-SY5Y cells with agoshRNA vectors, lipofectamine 2000 (Thermo Fisher Scientific) was used, following the manufacturer’s guidelines using 2 µg and 750 ng DNA for each transfection, respectively. For transfection of P19 cells with shRNA constructs, ViaFect Transfection Reagent (Promega) was used following the manufacturer’s guidelines. Two µg DNA was used for each transfection with a 1:3 ratio between DNA and transfection-reagent. At 48 h post-transfection, the cells were prepared for fluorescence-activated cell sorting (FACS; Online Resource 1). For HEK293T and SH-SY5Y cells, total RNA was harvested 48 h post-transfection and for SH-SY5Y cells in addition 8 days post-transfection.

### PAGE Northern Blot

PAGE Northern blot was performed as indicated in Online Resource 1.

### Lentiviral Production and Transduction

Lentiviral production and transduction was performed as indicated in Online Resource 1.

### RNA Extraction

From H9, HEK293T, and SH-SY5Y cells, total RNA was extracted with TRIzol Reagent (Invitrogen) using the manufacturer’s guidelines. RNA concentrations were measured using Nanodrop Lite (Thermo Fisher Scientific). From FACS-sorted P19 cells, total RNA was extracted using TRIzol reagent and PhaseMaker tubes (Invitrogen) following the manufacturer’s guidelines. RNA concentrations were measured using Nanodrop One (Thermo Fisher Scientific).

### Fluorescence-Activated Cell Sorting (FACS)

FACS was performed as indicated in Online Resource 1.

### cDNA Synthesis and RT-qPCR

Prior to cDNA synthesis, total RNA extracted from H9 cells was treated with DNase I (Thermo Fisher Scientific) according to the manufacturer’s protocol. cDNA for all RT–qPCR expression analyses was synthesized from total RNA by MLV-RT (Invitrogen) according to the manufacturer’s guidelines using random hexamer primers. RT–qPCR was performed with SuperScript III Platinum SYBR Green One-Step qPCR Kit (Thermo Fisher Scientific) on a LightCycler 480 (Roche) according to standard procedures, using either divergent primer sequences spanning the BSJ or primer pairs spanning an exon-exon junction (Table S3). For H9 samples, expression levels were normalized to the mean of human *GAPDH* and *SF3A1* or *GAPDH* alone. For P19 samples, expression levels were normalized to the mean of mouse *Gapdh* and *Rpl13a*.

### Total RNA-Sequencing

Total RNA was subjected to ribosomal RNA (rRNA) depletion using the Ribo-Zero rRNA Removal Kit (Human, Mouse, Rat; Epi-centre, WI, USA). Sequencing libraries were generated with the ScriptSeq v2 RNA-Seq Library Preparation Kit (Epicentre), quality controlled on the 2100 Bioanalyzer (Agilent Technologies), and quantified using the KAPA library quantification kit (Kapa Biosystems, MA, USA). RNA-Seq was performed using the HiSeq 4000 system (Illumina, San Diego, CA, USA) at the Beijing Genomics Institute (BGI) using the 100 bp paired-end sequencing protocol.

### NanoString Analyses

All NanoString experiments were performed using the NanoString nCounter SPRINT (NanoString Technologies, WA, USA) following the manufacturer’s instructions. For a detailed method, see Online Resource 1. Probes are listed in Table S4.

### QuantSeq 3’ End RNA Sequencing

Total RNA was isolated using TRIzol reagent (Invitrogen), RNA quality was controlled using the RNA 6000 Nano Kit (Agilent Technologies, CA, USA) on a Bioanalyzer 2100 (Agilent Technologies), and RNA quantity was determined using the Qubit RNA HS Assay Kit (Thermo Fisher Scientific) on a Qubit 4 Fluorometer (Thermo Fisher Scientific). The TURBO DNA-*free* Kit (Thermo Fisher Scientific) was used for DNA removal followed by an additional ethanol-based RNA precipitation step. For library preparation, the QuantSeq 3’mRNA-Seq Library Kit (Lexogen, Vienna, Austria) was utilized according to the manufacturer’s instructions at the Department of molecular medicine, Aarhus University Hospital, Denmark. For a detailed method, see Online Resource 1.

### Single-Cell RNA-Sequencing: Library Preparation

For the droplet-based scRNA-seq library preparation, earlier publications were followed [38, 40]. For a detailed method, see Online Resource 1.

### In UteroElectroporation (IUE)

Prior to surgery, E15.5 pregnant females were injected with a single analgesic dose of buprenorphine (0.05 mg/kg). After 30 min, gas anesthesia was induced with 5% isoflurane. After induction, isoflurane was kept at 1.5–2%. Abdominal fur was removed, and surgery was performed in sterile conditions. The laparotomy was initiated by a vertical incision in the skin of approximately 2.5 cm. The skin and muscle layers were separated, followed by a vertical cut through the abdominal muscle layer. Uterine horns were exposed and constantly kept moist with physiological saline. All embryos of one mother were injected intracerebroventricularly with 1.2 µl of a mix comprising 1.5 µg/µl plasmid (0.75 µg/µl pll3.7_shFat3_I and 0.75 µg/µl pll3.7._shRNA_circFat3_II or 1.5 µg/µl pll3.7._shRNA_Scr) and FastGreen in physiological saline. The surgeon was blinded for the construct. Correct injection was verified by the distribution of FastGreen through the ventricular system. After all embryos were injected, a tweezer cathode was placed laterally, and an additional single anode was placed frontally on the cortical midline. The electrodes were fired with 30 V for 50 ms, 5 times with an interval of 950 ms. After the electroporation of all embryos, the uterine horns were placed back into the abdomen. First, the muscle layer and, subsequently, the skin were closed with a continuous suture. For the prevention of infection and local pain, fucidine and lidocaine creams were administered on the wound. Gas anesthesia was terminated, and the mouse was transferred to a preheated cage (37 °C) for a few hours. For the following 2 days, systemic analgesics were given by administering carprofen in the drinking water.

### BaseScope Single Molecule RNA FISH

In situ hybridization (ISH) was performed using BaseScope technology (ACD, Newark, CA) with custom hybridization probes covering the BSJ of circFAT3 (1ZZ probe: BA-Hs-FAT3-E2-circRNA targeting 3633–369 of NM_001367949.2) or ciRS-7 (1 ZZ probe: BA-Hs-CDR1as-circRNA-Junc targeting TCTTCTGACATTCAGGTCTTCCAGTGTCTG-CAATATCCAGGGTTTCCGATGGCACCTGTGTCAAGGTCTTCCAACAACTC) following the instructions provided by the manufacturer (ACD#323,900). For a detailed method, see Online Resource 1.

### Immunostaining: Cerebral Organoids

For immunostaining procedures, COs were washed three times in PBS, fixed in 4% PFA at 4 °C for 20–60 min (dependent on CO size), and washed three times in PBS. After submerging the COs in 40% sucrose until they sunk, the tissue was embedded in 13%/10% gelatin/sucrose and frozen blocks stored at − 80 °C. Cryostat (Leica) sections were cut at a thickness of 12 µm. For embedding media removal, sections were placed into warm PBS for 15 min. Subsequently, a second fixation of 15 min with 4% PFA and three washing steps with PBS were performed. After blocking and permeabilizing the sections with 0.25% TritonX-100 (Sigma), 0.1% BSA (Sigma), and 5% normal donkey serum (Sigma) in PBS for 1 h, sections were incubated with primary antibodies: mouse anti-MAP2 (AMAb91375, Atlas antibodies, 1:1000), rabbit anti-SOX2 (AB5603, Millipore, 1:500), rabbit anti-GFAP (Z0334, DAKO, 1:1000), mouse anti-DCX (Sc-271390, Santa Cruz Biotechnology, 1:200), rabbit anti-Ki67 (RM-9106-S1, Thermo Scientific, 1:200), or mouse anti-TUJ1 (MAB1195, R&D, 1:1000) in blocking solution (0.1% Triton X-100, 0.1% BSA, 5% normal donkey serum in 1XPBS) at 4 °C overnight. Then, sections were washed three times in PBST (0.1% Triton X-100 in PBS). The tissue sections were incubated with secondary antibodies (anti-rabbit-647, A31573; anti-goat-546, A10036; anti-mouse-547, A11056; anti-mouse-647, A32728TR) at room temperature for 1 h and counterstained with DAPI (Thermo Fisher Scientific, 1 µg/ml) for 10 min. After three PBST washes, tissue was mounted using ProLong Gold antifade (Invitrogen), and images were acquired with Zeiss laser scanning confocal LSM800 or the Olympus VS120 Slide Scanner System with 20 × , 40 × , and 63 × objectives. Image processing and analysis were performed in Fiji [41], and quantification was performed as indicated in Online Resource 1.

### Immunostaining: IUE

Electroporated brains were harvested at P2, fixed in 4% PFA overnight and thoroughly washed with 1 × PBS. The brains were embedded in 4% low-temperature melting agarose in 1 × PBS and cut into 60 µm thick sections on a Leica VT1000S Vibratome. The free-floating sections were permeabilized and blocked with blocking solution (10% FCS and 1% Triton X-100 in 1 × PBS) for 2 h at room temperature and stained in primary antibodies overnight at 4 °C. Primary antibodies, chicken anti-GFP (GFP-1020, AVES Labs) and rat anti-Ctip2 (ab18465, Abcam), were incubated 1:500 in blocking solution. The next day, sections were washed with 1 × PBS, and incubated in secondary antibodies: Alexa Fluor 488 Donkey anti-chicken (703–545-155, Jackson ImmunoResearch), Alexa Fluor 647 Donkey anti-rat (ab 150,155, Abcam), and Alexa Fluor 568 Donkey Anti-Rabbit IgG H&L (ab175470) 1:500 in blocking solution for 2 h at room temperature. Next, the sections were immersed in DAPI for 20 min, washed thoroughly with 1 × PBS, transferred onto microscopic glass slides, and mounted using Mowiol 4–88. Images were taken on an AxioScope A1 epifluorescence microscope.

### Quantification of Migration Defect of IUE Brains

Image processing and analysis were performed in Fiji [41], and cells were quantified as indicated in Online Resource 1.

### NanoString Data Analysis

The raw data for the custom-designed panel was processed using the nSOLVER 4.0 software (NanoString Technologies, WA, USA), and data analysis was performed as indicated in Online Resource 1.

### Total RNA Sequencing: Computational Processing

The total RNA-seq data concerning rostral and caudal NPCs were quality controlled (Phred score 20), and the adapter was trimmed using trim_galore (v0.3.3). Filtered data were mapped to the human genome (hg19) using TopHat2, and mRNA quantification was done using featureCounts [42], a part of the Subread package (v1.6.1) using Ensembl release 71 gene annotations. mRNA expression levels were further normalized using the median of ratios method implemented in the DESeq2 [43] package in R (http://www.r-project.org). CircRNAs were quantified using a stringent version of the find_circ pipeline as previously described [10] and the CIRCexplorer [44] pipeline. All circRNA data analyses were based on the output from the stringent find_circ pipeline, and circRNA candidates supported by at least five BSJ-spanning reads on average per sample were defined as high-abundance circRNAs. Among these, all circRNA candidates, not detected by CIRCexplorer, were manually inspected to exclude obvious mapping artifacts as previously described [16]. Reads per million (RPM) were calculated by dividing the sequencing reads aligning across the particular BSJs by the total number of reads multiplied by 1 million. The circular-to-linear (CTL) ratios were calculated by dividing the number of reads spanning the particular BSJ by the average linear reads spanning the splice donor- and splice acceptor sites of the same BSJ plus one pseudo count (to avoid division by zero). Potential correlations were assessed by calculating Pearson’s correlation coefficients, *R*^2^, and assessing if the slopes were significantly non-zero with an *F*-test.

### QuantSeq 3’ End Sequencing: Computational Processing

Raw reads were filtered and trimmed using trim_galore (version 0.6.5dev, using parameters: -a AAAAAAAAAAAA –clip_R1 12). The filtered QuantSeq 3’ mRNA-Seq reads were mapped to the human reference genome (hg19) using STAR (version 2.7.3a) [45] with default setting. Read mapping results per gene were then quantified using featureCounts [42] with gene annotation from Gencode (version 28). Additionally, the MACS2 callpeak algorithm (with parameters: –nomodel –shift 0 -g 2e9) was used to identify high read-density regions in the genome. Differential expression analysis (DEA) was based on the featureCounts output using DESeq2 [43]. For subsequent KEGG pathway and GO-term analysis of differentially expressed genes (DEGs), the Database for Annotation, Visualization and Integrated Discovery (DAVID; v6.8) [46, 47] with a customized background of all detected genes with basemean > 5 was utilized. All plotting and graphs were either done in R or using Prism 9 (GraphPad, CA, USA).

### Single-Cell RNA-Sequencing: Computational Processing

#### Raw Data and Processing

Raw basecall images from the Illumina Nextseq500 machine were demultiplexed using Illumina’s bcl2fastq command line tool. Read-1 of the paired-end fastq files contained the cellular barcode (12 nucleotides) and the unique molecular identifier (UMI, 8 nucleotides), which is standard practice when sequencing a droplet-based single-cell library. Read-2 consisted of 64 nucleotides. Raw fastq files were processed with the help of Drop-seq_tools-2.3.0 (https://github.com/broadinstitute/Drop-seq) and picard-tools-2.21.6 (https://broadinstitute.github.io/picard/). The whole data processing was written in snakemake (https://snakemake.readthedocs.io/) and followed the standard Drop-seq pipeline steps. First R1 and R2 files were merged into a single.bam file. Then, each read was tagged with its own cell-barcode (CB) and UMI. Each read was polyA- and adapter trimmed. Reads were then mapped with STAR (2.7.1a) in single-end mode, and each read was tagged with a gene. After barcode repair (substitution errors and synthesis errors), a digital expression matrix was created (DGE) for each sample to be used for further downstream data analysis.

#### Clustering and Data Analysis

Clustering and data analysis was written in python using the scanpy package (https://scanpy.readthedocs.io/). Before clustering, cells with less than 200 UMI or more than 5% mitochondrial gene counts were removed. Only cells which expressed more than 10 genes and genes which were expressed in more than 3 cells were kept. Cells with more than 2500 genes were removed. Before conducting a principal component analysis (PCA), the top 2000 highly variable genes were identified, and the data was scaled to have 0 mean and unit variance. Then, the data were clustered using the Leiden algorithm [48], and the dimensionality was reduced with UMAP (https://arxiv.org/abs/1802.03426). After clustering, cell type markers were identified based on differentially expressed genes (*t*-test on cluster vs all the other cells). Cell types were then identified based on known markers from the literature. For the final clustering, the data were processed as above, but all cells having less than 500 UMI were removed. Also, stress-induced and necrotic clusters from the previous clustering were removed to regress out stress-related effects.

#### Marker Gene and Cell-Type Identification

After clustering, per-cluster marker genes were identified. To identify significantly enriched genes (for each cluster, using the rest of the clusters as background), we used the non-parametric Mann–Whitney *U* (or Wilcoxon-rank-sum) statistical test, using scanpy’s rank_genes_groups function. After identifying the top 20 markers per cluster, clusters were identified based on literature and marker-gene expression.

#### Differential Expression Analysis

For Figs. 4G and 5F, differentially expressed genes between selected clusters were identified with the DESeq2 [43] R package, using the likelihood ratio test as a hypothesis test instead of the default Wald test. Cells from each cluster were treated as independent replicates across two conditions (the two different clusters). To have more statistical power, only highly abundant cells (with more than 2000 and less than 4000 UMI) were selected as input for the analyses.

### NCBI Blast for Mature agoshRNAs

The mature guide strand RNAs were blasted against the NCBI Transcript Reference Sequences (refseq_RNA), using BLASTN [49] with standard parameters, but excluding models (XM/XP) and adjusted for short input sequences.

### Statistical Analyses

All statistical tests concerning the circRNA analyses were performed using Prism 7 (GraphPad, CA, USA). Comparison between the expression levels of the high-abundance circRNAs in the different sample groups was done using a Wilcoxon test as the data were not normally distributed according to the D’Agostino and Pearson normality test. All statistical tests concerning 2D and 3D in vitro cell cultures and murine in utero electroporation were performed using Prism 9 (GraphPad, CA, USA). Either a two-tailed unpaired *t*-test or a one-sample *t*-test on log2-transformed values for fold change comparisons was used. Details on statistical tests can be found in the specific method sections above.

## Results

### Global Increase of circRNA Expression upon Differentiation of Human Embryonic Stem Cells into Rostral and Caudal Neural Progenitors

**Fig. 1:**
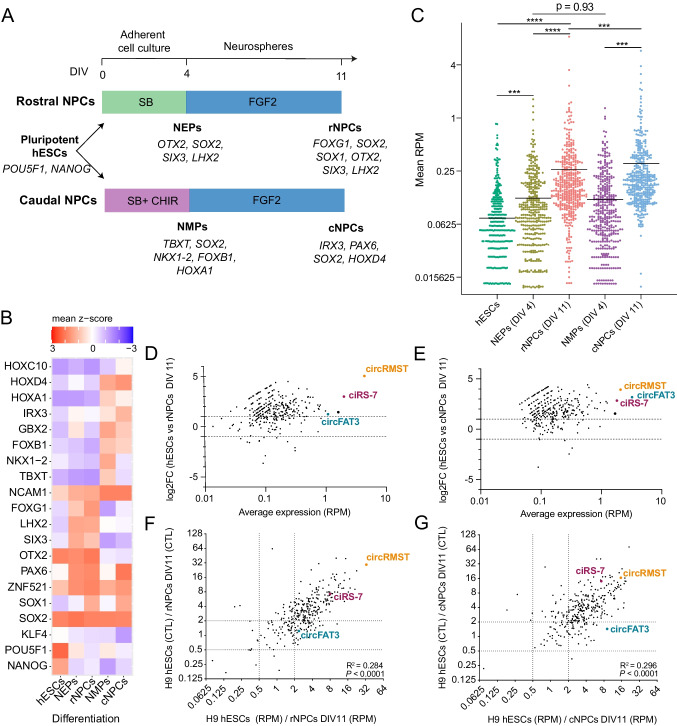
Global increase of circRNA expression upon differentiation of human embryonic stem cells into rostral and caudal neural progenitors. **A** Eleven-day differentiation protocol of H9 human embryonic stem cells (hESCs) into rostral and caudal neural progenitor cells (rNPCs/cNPCs) using SB431542 (SB) and SB plus CHIR99021 (CHIR), respectively, until day four and FGF2 as supplement between day 4 and 11. Differentiations were performed in biological triplicates. **B** Heatmap showing expression levels of characteristic markers in hESCs, NEPs, rNPCs, NMPs, and cNPCs [35] that were log2-transformed (normalized counts + pseudocount) and converted to a *z*-scale; *n* = 3. **C** Column scatter plot of 417 high-abundance circRNAs detected in hESCs, NEPs, rNPCs, NMPs, and cNPCs; *n* = 3. Wilcoxon matched-pairs signed rank test, ***p < 0.001, ****p < 0.0001. **D**, **E** MA plots comparing the 417 high-abundance circRNAs between hESCs and rNPCs (**D**) as well as hESCs and cNPCs (**E**). Log2 fold changes, and average expression were plotted; *n* = 3. **F**, **G** Scatter plots of fold changes in RPM and fold changes in circular-to-linear (CTL) ratios of the 417 high-abundance circRNAs for hESCs versus rNPCs (**F**) and hESCs versus cNPCs (**G**); *n* = 3. Correlations were assessed by calculating Pearson’s correlation coefficients, *R*^2^, followed by an *F*-test. RPM, reads per million; DIV, days in vitro

To assess potential roles of circRNAs in neural development, we first characterized the expression landscape of circRNAs in a 2D model during early neurogenesis. H9 human embryonic stem cells (hESCs) were differentiated into neuroepithelial or -mesodermal progenitor cells (NEPs/NMPS) and further into rostral and caudal neural progenitor cells (rNPCs and cNPCs, respectively) for 11 days, as previously described [35] (Fig. 1A). The total RNA from days 0, 4, and 11 was subjected to RNA-seq, and proper cell differentiation was confirmed by the expression of lineage-specific genes (Fig. 1A, B). As expected, hESCs expressed high levels of the stem cell markers *NANOG* and *POU5F1*, while neural progenitor cells expressed *SOX2*. NEPs showed early rostral markers (*OTX2*, *SIX3*, and *LHX2)*, which continued to be present in the rNPCs in addition to late rostral markers (*SOX1* and *FOXG1)*. In contrast, NMPs were characterized by early caudal markers (*TBXT*, *NKX1-2*, *FOXB1*, and *HOXA1)* in the absence of *PAX6* [50], while the caudal identity of cNPCs was confirmed with the late marker *IRX3* as well as other *HOX* genes.

Next, we analyzed the total RNA-seq data for circRNA expression and detected 8738 unique circRNAs, using a stringent version of the find_circ algorithm [10]. Among these circRNAs, 6911 (79.1%) were present in circBase [51], and 6984 (79.9%) were also detected by CIRCexplorer [44]. As circRNAs supported by very few reads are less likely to have biological relevance [52], we focused on circRNAs with an average of at least five BSJ-spanning reads in all samples (> 75 BSJ-spanning reads in total). This resulted in 417 circRNAs, which were analyzed further (Figure S1A). Based on the expression of these abundant circRNAs, the different cell types could clearly be distinguished in PCA (Figure S1B), and circRNA expression generally increased during differentiation (Fig. 1C). Among the 417 high-abundance circRNAs, 220 and 245 were upregulated more than two-fold during differentiation in rNPCs and cNPCs compared to hESCs, respectively, whereas only 12 and 5 were downregulated, respectively (Fig. 1D, E). In addition, increased CTL ratios were observed during differentiation for most circRNAs (Fig. 1F, G), suggesting a preferential production and/or accumulation of the circular spliced forms.

We selected three of the most strongly upregulated and highly expressed circRNAs (Fig. 1D, E and Figure S1C) for further functional analyses: circRMST, circFAT3, and ciRS-7. CircFAT3 was upregulated along with its linear host gene in cNPCs, whereas circRMST [53] and ciRS-7 [8, 9, 54, 55] showed more independent expression from their respective host gene (Fig. 1F, G and Figure S1D).

Taken together, we found a global upregulation of circRNAs during early neural differentiations in accordance with earlier publications [12, 53], including the transcripts circRMST, circFAT3, and ciRS-7.

### Individual circRNAs Can Be Efficiently Depleted with AGO-Dependent Short Hairpin RNAs

To enable loss-of-function studies for ciRS-7, circRMST, and circFAT3, we developed AGO-dependent short hairpin RNAs (agoshRNAs) [56, 57] designed to target their BSJs (Figure S2A; Table S1). AgoshRNAs are processed by Argonaute 2 (AGO2) and further trimmed by the poly(A)-specific ribonuclease (PARN) which eliminates off-targeting by passenger strands [57, 58]. To validate the proper processing of the agoshRNAs, we performed PAGE-Northern blots with probes targeting the mature guide strand RNAs (Table S2) and found the expected products for all three agoshRNAs [58] (Figure S2B).

**Fig. 2:**
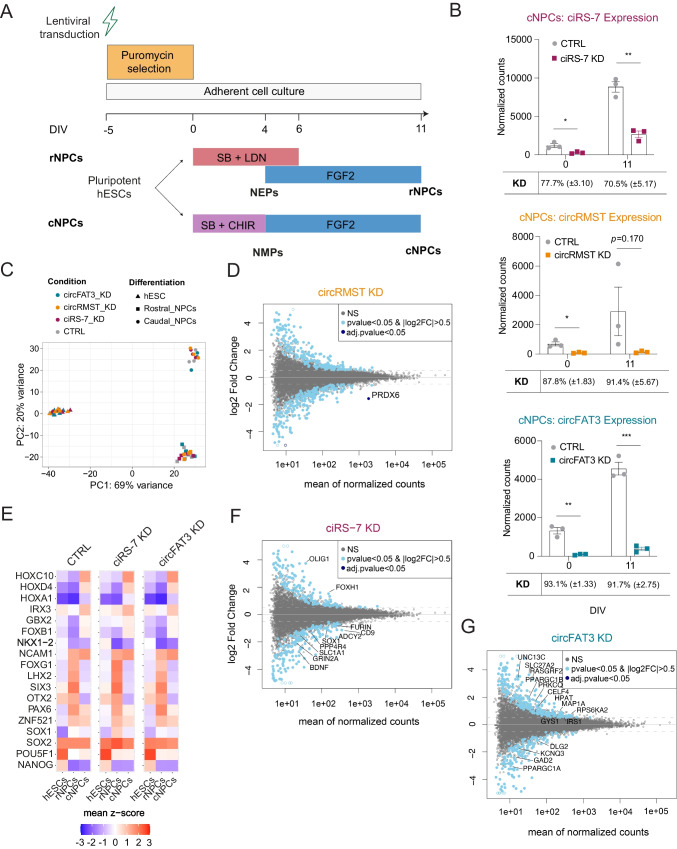
Depletion of circFAT3 leads to minor gene expression changes in early neural differentiation. **A** Pluripotent hESCs were transduced five days prior to differentiation with agoshRNA constructs or empty agoshRNA expression vector as control (CTRL). Transduced cells were selected with puromycin until differentiation onset. For rNPCs, SB and LDN193189 were used from day zero to 6 as well as FGF2 from day 4 to 11. For cNPCs, SB and CHIR were used from day zero to four, and FGF2 from day 4 to 11. Differentiations were performed in biological replicates. **B** CircRNA quantification using NanoString nCounter technology. ciRS-7, circRMST, and circFAT3 expression as normalized counts in KD versus CTRL cNPCs; *n* = 3. Mean values per time point ± SEM are shown. Two-tailed unpaired *t*-tests, ns: not significant, *p < 0.05, **p < 0.01, ***p < 0.001. **C** Principal component analysis (PCA) of CTRL, circRMST KD, circFAT3 KD, and ciRS-7 KD human embryonic stem cells (hESCs; *n* = 6), rostral neural progenitor cells (rNPCs; *n* = 4), and caudal NPCs (cNPCs; *n* = 3). **D** MA plots depicting global changes in differentially expressed genes (DEGs) in circRMST KD based on DESeq2 analysis. Plotted are average expression across samples and log2 fold changes. **E** Profiling of days 0 and 11 differentiated H9 hESCs using characteristic markers established in Fig. 1B and as previously described [35]. Normalized DESeq2 counts were log2-transformed (normalized counts + pseudo counts) and converted to a z-scale. **F**, **G** MA plots depicting global changes in differentially expressed genes (DEGs) in ciRS-7 KD (**F**) and circFAT3 KD (**G**) based on DESeq2 analysis. Plotted are mean expressions against log2 fold changes. KD, knockdown; DIV, days in vitro

Five days prior to the onset of differentiation into rNPCs and cNPCs, H9 hESCs were transduced with lentiviral vectors encoding agoshRNAs or control vectors and subsequently selected with puromycin (Fig. 2A).

To validate knockdown (KD) efficiency, we initially focused on the cNPC differentiation. We measured ciRS-7, circRMST, and circFAT3 levels in agoshRNA-transduced cells using NanoString nCounter analysis [59] at days 0, 4, and 11 and compared to cells transduced with control vector (CTRL). Efficient KD of all three circRNAs was observed in both hESCs and cNPCs (Fig. 2B), which was subsequently confirmed by RT-qPCR in both cNPCs and rNPCs (Figure S2C,D). We also confirmed that linear *FAT3* expression was not significantly altered upon circRNA KD (Figure S2E). While linear *RMST* also showed no significant changes upon circRNA KD before differentiation (Figure S2F), the high variation in expression levels at day 11 between replicates precluded firm conclusions for this timepoint.

### Depletion of circFAT3 Leads to Minor Gene and miRNA Expression Changes in Early Neural Differentiation

To investigate how circRNA depletion affects global mRNA levels during differentiation, we subjected total polyadenylated RNA from hESCs as well as rNPCs and cNPCs (circRNA KD and CTRL) to 3’-end sequencing. Data from CTRL cells showed that hESCs, rNPCs, and cNPCs could clearly be distinguished based on their mRNA expression patterns in PCA (Fig. 2C).

Next, we performed mRNA DEA comparing the circRNA KD cells with CTRL cells to distinguish common genes functioning in pathways for hESCs, rNPCs, and cNPCs (Online Resource 2). To rule out possible off-target effects, we compared the list of DEGs to a list of possible off-target genes for each of the three agoshRNAs, generated using BLAST [49] (Online Resource 3). For circRMST KD, we observed a DEG that coincided with a putative off-target gene, namely *PRDX6* (Fig. 2D). To test if the altered *PRDX6* expression was a real off-target effect of the agoshRNA, we transfected the circRMST agoshRNA-expressing vector into SH-SY5Y cells, which do not express circRMST. Again, we observed a downregulation of *PRDX6* (Figure S2G), indicating that this is indeed an off-target effect, and we therefore excluded circRMST KD cells from further analyses.

To test the effect of circRNA depletion on neuronal differentiation, we first verified that the KD cells undergo proper differentiation using the same lineage markers as in Fig. 1B. In general, the expression patterns were similar in KD and CTRL cells (Fig. 2E), indicating that neither ciRS-7 nor circFAT3 depletion abolished early neurogenesis or altered early patterning. Similarly, we did not find significant genes (adj. *p*-value < 0.05) upon depletion of ciRS-7 or circFAT3. However, when considering hits that did not pass correction for multiple testing, we found 239 and 272 DEGs, respectively (*p*-value < 0.05; basemean (BM) > 10; |log2FC|> 0.5) (Fig. 2F, G). For ciRS-7 and circFAT3 KD, genes involved in the synaptic organization (e.g., *BDNF*, *SLC1A1)* and synaptic signaling (e.g., *CELF4*, *DLG2*, *MAP1A*, *UNC13C)* were altered, respectively (Fig. 2F, G, and Figure S2H). In addition, for circFAT3 KD, genes related to insulin signaling and insulin resistance (*PPARGC1A*, *SLC27A2*, *PPARGC1B*, *PRKCQ*, *GYS1*, and *IRS1*; Fig. 2G) were the top hits. However, none of these pathways passed the false discovery rate (FDR) cut-off (Online Resource 4).

It has been shown previously that circRNAs may regulate miRNA expression levels through RNA-RNA interactions [8, 9, 15, 16]. Hence, to predict miRNA binding sites within ciRS-7 and circFAT3, we used circInteractome [60] (Figure S3A). In addition, to establish whether the level of circFAT3 and ciRS-7 could influence miRNA expression, we performed miRNA profiling using NanoString nCounter miRNA panel targeting 799 human miRNAs in CTRL as well as KD hESC, rNPCs, and cNPCs. Initially, we validated the expression of lineage-specific miRNAs in the CTRL cells, and as expected, stem cell-specific miRNAs, including the miR-302 cluster and miR-367-3p [61], were highly expressed in hESCs (Figure S3B). In addition, miR-10b-5p and miR-219a-2-3p were increased in cNPCs, while miR-204-5p was upregulated in rNPCs, as previously described [62].

miRNA profiling in the ciRS-7 KD cells compared to CTRL identified 4 and 19 differentially expressed (DE) miRNAs in rNPCs and cNPCs, respectively (*p*-value < 0.05, BM > 10; |log2FC|> 0.5) (Figure S3C,E), of which only six passed correction for multiple testing. However, none of these miRNAs showed predicted binding sites for ciRS-7 (Figure S3A). Although ciRS-7 displays more than 70 binding sites for miR-7, no changes were detected in miR-7 expression levels upon ciRS-7 KD in either of the NPC populations, likely due to developmental stage-dependent low expression (Online Resource 5).

For circFAT3 KD, 18 and 5 DE miRNAs were found in rNPCs and cNPCs, respectively (Figure S3D, F), of which none passed the FDR cut-off. Also, none of the DE miRNAs were shared between cNPCs and rNPCs.

Taken together, despite the efficient depletion of ciRS-7 and circFAT3 in hESCs and early NPCs, we only detected subtle gene expression changes using bulk RNA-seq on a heterogenous progenitor population. Similarly, only minor miRNA changes were found to be significant after adjusting for multiple testing upon ciRS-7 and circFAT3 depletion.

### Establishment of Cerebral Organoids to Study circRNA Function During Later Stages of Human Brain Development

**Fig. 3:**
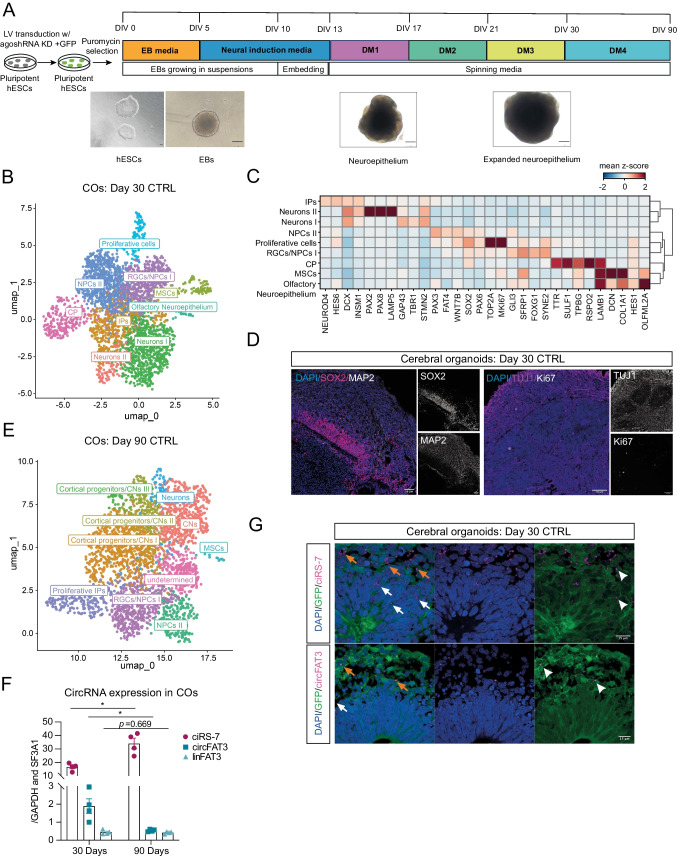
Establishment of cerebral organoids (COs) to study circRNA function during later stages of human brain development. **A** Generation of COs based on previous publications [36, 37]. After embryoid body (EB; DIV 2–5) generation from transduced and puromycin-selected hESCs, media was changed to neural induction media until day 13. EBs were grown in suspension for 10 days, before embedding them in Matrigel. From day 13, COs were cultured in spinning media. Differentiation media 1 (DM1) was supplemented with CHIR for 3 days [36]. After day 30, DM4 was additionally supplemented with BDNF, GDNF, cAMP, and TGFβ [37]. Differentiations were performed as two individual experiments. DIV, days in vitro; scale bar = 200 µm. **B** UMAP plot of scRNA-seq of day 30 CTRL COs (*n* = 2). Clustering of 3095 cells (with > 500 UMI) into nine clusters. NPCs, neural progenitor cells; RGCs, radial glial cells; IPs, intermediate progenitor cells; CPs, choroid plexus cells; MSCs, mesenchymal cells. **C** Heatmap of selected genes (depicted as mean *z*-score across cells within a cluster) characterizing the nine clusters identified in **B**. **D** IHC of day 30 CTRL COs. Upper panel: grey = MAP2, magenta = SOX2, blue = DAPI; Lower panel: pink = TUJ1, grey = Ki67. 12 µm cryostat sections; scale bar = 50 µm. **E** UMAP plot of scRNA-seq of day 90 CTRL COs (*n* = 2). Clustering of 2727 cells (with > 500 UMI) into nine clusters. **F** CiRS-7, circFAT3, and linear *FAT3* levels in days 30 and 90 CTRL COs were determined via RT-qPCR. Plots show values as mean ± SEM. Mean (ciRS-7; day 30) = 16.7 ± 2.8; mean (ciRS-7; day 90) = 34.0 ± 7.7; mean (circFAT3; day 30) = 1.89 ± 0.82, mean (circFAT3; day 90) = 0.54 ± 0.06; mean (FAT3; day 30) = 0.45 ± 0.12 and mean (FAT3; day 90) = 0.42 ± 0.06; normalized to the mean of *GAPDH* and *SF3A1*; *n* = 4 from two CO batches; two-tailed unpaired *t*-test, *p < 0.05. **G** Cell nuclei were stained with DAPI (blue), cell cytoplasm GFP^+^ (green) through agoshRNA expression vector, and circRNA ISH (magenta). Upper panel: ciRS-7 probe. Lower panel: circFAT3 probe. White arrows indicate circRNA expression outside of germinal zone. Orange arrows indicate circRNAs within the germinal zone. White arrowheads showing subcellular localization of circRNAs. 12 µm cryostat sections; scale bar = 15 µm

Due to the short-term differentiation and limitations in bulk analyses on heterogeneous cell populations in our 11-day differentiations, we explored the consequences of ciRS-7 and circFAT3 KD at later stages of human neurogenesis using a more complex human in vitro model. We differentiated COs from CTRL- and agoshRNA-expressing H9 cells following previous publications [36, 37, 63] (Fig. 3A) and initially used scRNA-seq to identify the cell type composition in CTRL COs at days 30 and 90 of differentiation (Fig. 3B and E, respectively).

At day 30, a total of nine unique cell type clusters could be detected equally between replicates (Figure S4A), including telencephalic radial glial cells (RGCs/NPCs I), non-telencephalic NPCs (NPCs II), proliferative cells (G2M/S), intermediate progenitors (IPs), and two clusters of neurons (Fig. 3B, C). RGCs/NPCs I were characterized by forebrain-specific markers (*PAX6* and *FOXG1*) together with *GLI3* and *SOX2* [64], while choroid plexus (CP) cells were defined by *TTR* and *SULF1* co-expression (Fig. 3C). *PAX6* expression was seen in both NPC clusters and proliferative cell types, whereas the second NPC cluster of non-telencephalic origin (NPCs II) showed less *PAX6* expression, lacked *FOXG1* expression, and contained cells expressing *CXCR4* [65], *PAX3*, and *PAX7*, which indicated the presence of non-telencephalic NPCs likely of mesencephalic/metencephalic identity. Proliferative cells showed a characteristic *TOP2A* and *MKI67* gene marker pattern [66] and neurons as well as neuronal progenitor clusters could be distinguished by augmented *STMN2, DCX,* and *GAP43* levels [65]. The Neurons II cluster showed a high abundance of *PAX2* and *PAX8*, which indicated the presence of an early neuronal cell type with the non-telencephalic identity of the midbrain-hindbrain boundary and further underpinned the presence of a non-telencephalic NPC population [67, 68]. Additionally, we observed *TBR1*-positive early-born cerebral cortical neurons and *EOMES*, *INSM1*, *HES6*, and *NEUROD4-*positive IP populations [64] (Fig. 3C). Immunostaining of day 30 CTRL COs identified SOX2-positive primary neural progenitor cells that generate ventricle-like structures (Fig. 3D), which were surrounded by early TUJ1-, DCX-, and mature MAP2-positive neurons.

In day 90 CTRL COs, we identified ten unique cell types using scRNA-seq, among others telencephalic RGCs/NPCs, non-telencephalic NPCs, and five clusters of neurons, four of which contained cortical progenitors or neurons (CNs) (Fig. 3E). Here, early *SOX2*-positive NPCs (NPCs II) appeared with an astrocyte-like profile, distinguished by increased expression of *GFAP*, *CLU*, and *S100B* [66] (Figure S4B,C). Day 90 neuronal cell types showed a broad TUJ1- and MAP2-immunoreactivity (Figure S4C,D) and appeared with a transcriptional profile corresponding to their localization within the cortical layers, e.g., with *CUX2* and *KCNQ3* in upper layer cortical progenitors (layer II-IV), layer II-V characteristic *SATB2* expression, and *LMO3* (subplate) in cortical neurons [69] (Figure S4B).

RT-qPCR analysis of ciRS-7, circFAT3, and linear *FAT3* levels in CTRL COs at days 30 and 90 (Fig. 3F) showed that ciRS-7 expression increased from day 30 to 90, circFAT3 expression declined in the same period, whereas linear *FAT3* remained unchanged. Notably, circFAT3 was on average lower expressed than ciRS-7 and four-times more abundant than linear *FAT3* in day 30 COs.

We also determined the subcellular localization of circFAT3 and ciRS-7 in CTRL COs using BaseScope ISH, which revealed that both circRNAs were expressed in differentiated cells and less abundant in cells in the germinal zone forming the ventricle-like structures (Fig. 3G). As reported previously [70–72], the majority of circRNAs were found in the cytoplasm, often in a peri-nuclear localization (Figure S4E,F).

In summary, we characterized the cell type composition and circRNA expression profile in CTRL COs and identified a ciRS-7 upregulation from day 30 to day 90 of differentiation, while circFAT3 levels declined during the same time frame independently of linear *FAT3* expression.

### Depletion of circFAT3 Alters Abundance of Forebrain Radial Glial Cells in Day 30 Cerebral Organoids

**Fig. 4:**
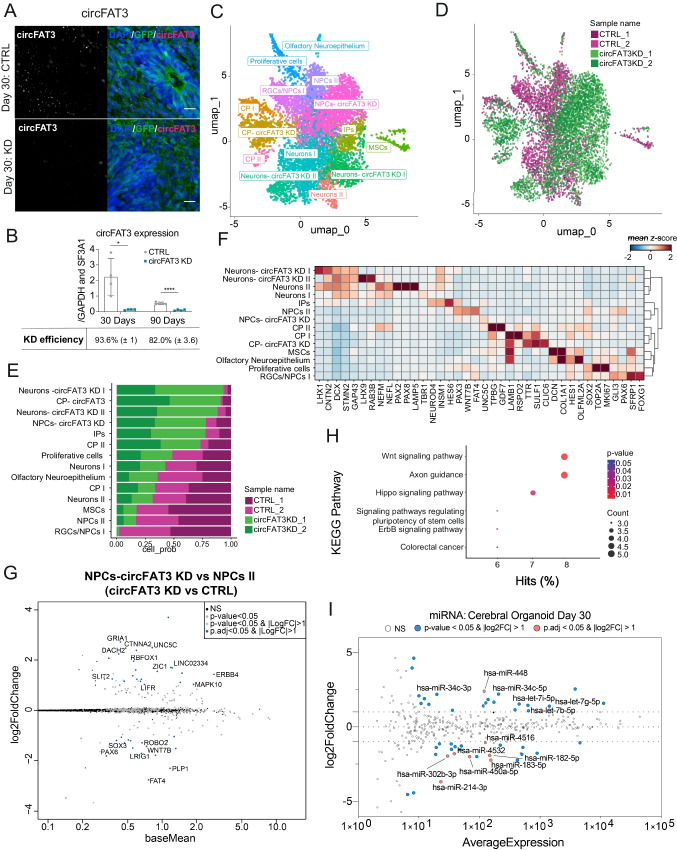
Depletion of circFAT3 alters abundance of forebrain radial glial cells in day 30 cerebral organoids (COs). **A** CircFAT3 ISH using BaseScope in day 30 COs. 12 µm cryostat sections; scale bar = 20 µm. **B** Quantification of circFAT3 KD using RT-qPCR. Expression was normalized to the mean of *GAPDH* and *SF3A1*. Plot shows mean ± SEM; *n* = 4 from two CO batches. Two-tailed unpaired *t*-test, *p < 0.05, ****p < 0.0001. **C** UMAP plot of scRNA-seq data from CTRL and circFAT3 KD COs at day 30 (*n* = 2) clustering of 3051 and 4166 cells (with > 500 UMI), respectively. When combining both conditions, 13 clusters were identified. NPCs, neural progenitor cells; RGCs radial glial cells; IPs, intermediate progenitor cells; CPs, choroid plexus cells; MSCs, mesenchymal cells. **D** UMAP plot of scRNA-seq data of day 30 COs demonstrating cell origin from either CTRL or circFAT3 KD in biological duplicates. Cell position corresponds to panel **C**. **E** Quantification of cell numbers per cluster derived from either CTRL or circFAT3 KD day 30 COs. Clusters defined as indicated in **C**. **F** Heatmap of selected genes (depicted as mean *z*-score across cells within a cluster) characterizing the 13 clusters identified in **C**. **G** MA plot showing differentially expressed genes (DEGs) identified between circFAT3 KD- and CTRL-specific non-telencephalic NPCs using DESeq2. Log2 fold changes are plotted relative to the mean expression. **H** KEGG pathway analysis of DEGs identified between circFAT3 KD- and CTRL-specific non-telencephalic NPCs. **I** MA plot showing DE miRNAs identified in bulk RNA in day 30 circFAT3 KD COs (*n* = 3). Average expression is plotted against log2 fold changes

To study the effects of ciRS-7 and circFAT3 in COs, we performed KDs using the same agoshRNA constructs as described above. The KD efficiency was quantified using RT-qPCR and confirmed by BaseScope ISH (Fig. 4A, B and Figure S5A, B) and while circFAT3 KD was very efficient (Fig. 4A, B), no major changes were seen in linear *FAT3* abundance (Figure S5C). For ciRS-7, a relatively modest KD was observed (Figure S5B), presumably due to the exceptionally high abundance of ciRS-7 in neuronal tissues. Therefore, we decided to characterize the circFAT3-depleted COs further.

To directly compare the transcriptional profiles of single cells originating from CTRL and circFAT3 KD COs, we performed a combined scRNA-seq cluster analysis. After 30 days of differentiation, we found 14 distinct cell clusters accounted for by CTRL and circFAT3 KD COs in combination (Fig. 4C). Although nine of the clusters corresponded in their transcriptional profile to clusters found previously in CTRL cells only (Fig. 3B), we noticed that cell type proportions within each cluster changed substantially between CTRL and circFAT3 KD COs (Fig. 4D, E). Clusters with more than 75% of cells originating from CTRL COs included mesenchymal cells (MSCs) and two distinct types of NPCs (RGCs/NPC I and NPC II), both with moderate to high abundance of *GLI3*, *PAX6*, and *SOX2* transcripts distinguished by *FOXG1* and *CXCR4/PAX3*, respectively (Fig. 4E, F). Consequently, upon circFAT3 KD, the number of MSCs and *FOXG1*-positive RGCs diminished markedly compared to CTRL COs. On the other hand, the proportion of some cell populations was strongly increased upon circFAT3 KD. Here, clusters with more than 75% of the cells deriving from circFAT3-depleted COs included non-telencephalic NPCs, two types of neurons, CP cells, and IPs. CircFAT3 KD COs included a unique NPC type (NPCs-circFAT3 KD) that resembled the transcriptional profile of CTRL non-telencephalic NPCs (NPCs II) (Fig. 4F). When directly comparing the two cell clusters, we identified 62 DEGs (adj. *p*-value < 0.05 and |log2FC|> 0.5, Online Resource 6) (NPCs- circFAT3 KD vs NPCs II in Fig. 4G)*.* Among others, we observed a downregulation of *PAX6*, *WNT7B*, and *LRIG1*. Additionally, we found a significant decrease in the protocadherin member *FAT4*, which has been associated previously with aberrant cortical migration, neuronal progenitor accumulation, and a reduction of neuronal differentiation [73, 74]. At the same time, we found several genes to be upregulated in circFAT3 KD-specific NPCs including *ERBB4*, *UNC5C*, *DCC*, and αN-catenin *CTNNA2* (Fig. 4G). Of note, besides *ERBB4* no other ventral telencephalic markers were found to be differentially expressed in these clusters. The deregulation was further confirmed on a subset of genes using RT-qPCR on bulk RNA from day 30 CTRL and circFAT3-depleted COs (Figure S5D).

We subjected the DEGs in circFAT3 KD-specific NPCs versus CTRL NPCs (adj. *p*-value < 0.05 and |log2FC|> 0.5) to KEGG pathway analysis and found the highest scoring pathways to be axon guidance, Wnt signaling, and Hippo signaling (Fig. 4H; Online Resource 7). Moreover, GO analysis revealed several categories of biological processes including negative regulation of neurogenesis, telencephalon development, axonogenesis, neuronal migration, and gliogenesis (Figure S5E).

We investigated DEGs between the populations of neurons derived from CTRL COs (Neurons I) and circFAT3 KD (Neurons- circFAT3 KD II) and found the expression of 35 genes to be altered (Figure S5F; adj. *p*-value < 0.05 and |log2FC|> 0.5, Online Resource 6), including an upregulation of *INSM1*, *DCC*, *LHX2*, and *LHX9* and downregulation of *NEFM* and *NEFL* (Fig. 4F and Figure S5F). For pathway analysis, no significant results could be obtained, while GO analysis showed noradrenergic neuron differentiation as a top hit for GO:biological processes (Online Resource 7).

Taken together, our data show an essential loss of forebrain RGCs and a decrease in MSCs in circFAT3-depleted COs, suggesting that circFAT3 plays a role in the generation of anterior cell populations and neuronal differentiation. In addition, we found hints towards circFAT3 involvement in the modulation of axon guidance and Wnt signaling in NPC populations.

### CircFAT3 Depletion Leads to miRNA Expression Changes in Day 30 Cerebral Organoids

To detect changes in miRNA expression based on alterations in cell type composition or circRNA modulation, we performed miRNA profiling using NanoString technology on CTRL and circFAT3-depleted day 30 COs (Fig. 4I, Online Resource 8). This showed nine DE miRNAs (adj. *p*-value < 0.05 and |log2FC|> 0.5), including miR-183 and miR-34c, which showed a two-fold downregulation and two-fold upregulation in circFAT3-depleted samples, respectively (Fig. 4I). This was in accordance with our 2D in vitro results that also showed a tendency for miR-183 downregulation in circFAT3 depleted cNPCs, as well as an upregulation of miR-34c in circFAT3 KD cells (Figure S3D,F). In circFAT3 KD COs, we detected an additional downregulation of miR-182, miR-302b, and miR-302c. The decrease in the latter two miRNAs, which are lowly expressed in cNPC in comparison to hESCs and rNPCs, was another indication of a high abundance of cNPCs in circFAT3-deficient COs (Figure S3B).

### Depletion of circFAT3 Leads to Shift in Neuronal Progenitor Cell State and Loss of Mature Cortical Neuron-Populations in Day 90 Telencephalic Cerebral Organoids

**Fig. 5:**
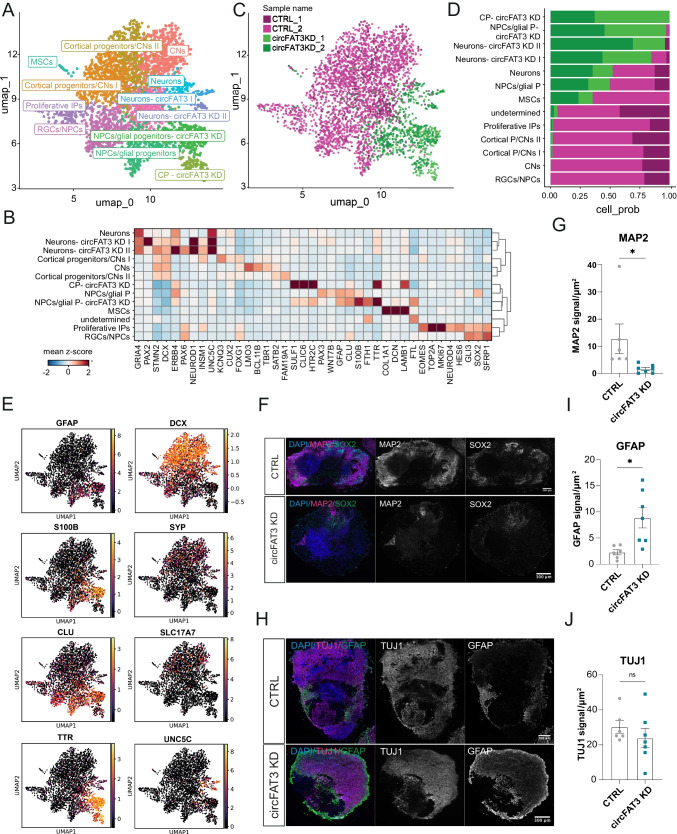
Depletion of circFAT3 leads to loss of neuronal progenitor- and mature cortical neuron-populations in day 90 telencephalic cerebral organoids (COs). **A** UMAP plot of scRNA-seq data of CTRL and circFAT3 KD-derived cells of day 90 COs (*n* = 2) clustering of in total of 2714 and 739 cells (cells with > 500 UMI), respectively. Combining both conditions resulted in the identification of 13 clusters in total. NPCs, neural progenitor cells; RGCs, radial glial cells; IPs, intermediate progenitor cells; CPs, choroid plexus cells, CNs, cortical neurons; MSCs, mesenchymal cells, P, progenitors. **B** Heatmap of selected genes (depicted as mean *z*-score across cells within a cluster) characterizing the 13 clusters identified in panel **A**. **C** UMAP plot of scRNA-seq data from CTRL and circFAT3 KD-derived cells of day 90 COs demonstrating cell origin from either CTRL or circFAT3 KD condition in biological duplicates. Cell position corresponds to cells in panel **A**. **D** Quantification of cell numbers per cluster derived from either CTRL or circFAT3 KD day 90 COs. Clusters defined as indicated in **A**. **E** Log-normalized expression of selected marker genes from A to B, showing per-cluster specificity and abundance per cell for each gene. **F** IHC images of day 90 CTRL and circFAT3-depleted COs. MAP2 = magenta; SOX2 = green; DAPI = blue. 12 µm cryostat sections; scale bar = 300 µm. **G** Quantification of MAP2 signals in day 90 CTRL and circFAT3 COs. The signal intensity was normalized to the area analyzed (µm^2^). Values are presented as mean ± SEM. *N* = 6 (CTRL) and *n* = 7 (circFAT3) from two CO batches. Two-tailed unpaired *t*-test, *p < 0.05. **H** IHC images of day 90 CTRL and circFAT3 COs. TUJ1 = magenta; GFAP = green; DAPI = blue. 12 µm cryostat sections; scale bar = 300 µm. **I**, **J** Quantification of GFAP (**I**) and TUJ1 (**J**) signals in day 90 CTRL and circFAT3 COs. The signal intensity was normalized to the area analyzed (µm^2^). Values are presented as mean ± SEM. *N* = 6 (CTRL) and *n* = 7 (circFAT3) from two CO batches. Two-tailed unpaired *t*-test, *p < 0.05, ns: not significant

To establish the effect of circFAT3 KD at a later time point, we profiled circFAT3 KD and CTRL COs after 90 days of differentiation using scRNA-seq and found 13 distinct cell clusters in a combined UMAP (Fig. 5A, B). Although we identified several cell types corresponding to our previous cell cluster definition for CTRL day 90 COs (Fig. 3E), the proportion of CTRL- and circFAT3 KD-derived cells within each cluster varied substantially (Fig. 5C, D). We observed a neuronal cell cluster (Neurons- circFAT3 KD I and II) and a distinct NPC/glial progenitor cell cluster (NPCs/glial P- circFAT3 KD) were only found in circFAT3 KD COs, whereas cortical progenitors, CNs, telencephalic RGCs/NPCs, and proliferative IPs were primarily present in CTRL tissue (Fig. 5D). Comparing the circFAT3 KD- specific NPCs with glial signature (NPCs/glial P-circFAT3 KD) with the CTRL-specific equivalent (NPCs/glial P), we saw an increase in *GFAP*, *S100B*, *CLU*, and CP marker *TTR*, as well as a decrease in the telencephalic marker *FOXG1* (Fig. 5B, E). However, we found no overall changes in *SOX2* and *NES* levels across conditions (Figure S6A-D), suggesting no overall loss of NPCs, but rather a shift in transcriptional state. The neurons predominantly found in circFAT3 KD COs (Neurons-circFAT3 KD I and II) were defined by their co-expression of neuronal markers, such as *DCX* and *STMN2* with a high abundance of the early neuronal marker *INSM1* and glutamatergic gene *GRIA4* (Fig. 5B, E). In addition, *UNC5C* and *ERBB4* were upregulated in the circFAT3 KD-specific neuron clusters. The reduction of neuronal markers, including *DCX* and *SYP*, and an increase in the expression of *GFAP*, *S100B*, *TTR*, and *SULF1* were validated in circFAT3 KD using RT-qPCR on bulk RNA (Figure S6A, B).

Next, to assess whether the differences found were reflected in protein expression, we performed immunofluorescent stainings that showed a loss of MAP2 for circFAT3 KD compared to CTRL COs, which indicates the absence of mature neurons (Fig. 5F, G). Also, we identified an increase of GFAP levels in circFAT3 KD COs at day 90, while only a subset of cells showed GFAP expression in day 90 CTRL COs (Fig. 5H, I). Finally, circFAT3 KD samples showed a high abundance of beta-III-tubulin (TUJ1)-positive cells (Fig. 5H, J), a general characteristic of postmitotic neurons from an early neuronal stage, without displaying co-labelling between GFAP and TUJ1 (Figure S6E). This suggests the presence of early neuronal progenitors in circFAT3 KD COs, but a deficit in mature MAP2-positive neuronal populations.

As expected from our day 30 COs data, circFAT3 depletion led to a loss of cortical neurons and forebrain-specific neuronal progenitors. However, the presence of CP cells and a small population of non-telencephalic *ERBB4*-, *GRIA4*-, and *UNC5C*-positive neurons were circFAT3 KD-specific. Overall, this indicates that circFAT3 affects several steps of neuronal development including differentiation and migration.

### In Utero Electroporation of Murine Prefrontal Cortex Unravels Developmental Defects for Neural Progenitors Lacking circFat3 Expression

**Fig. 6:**
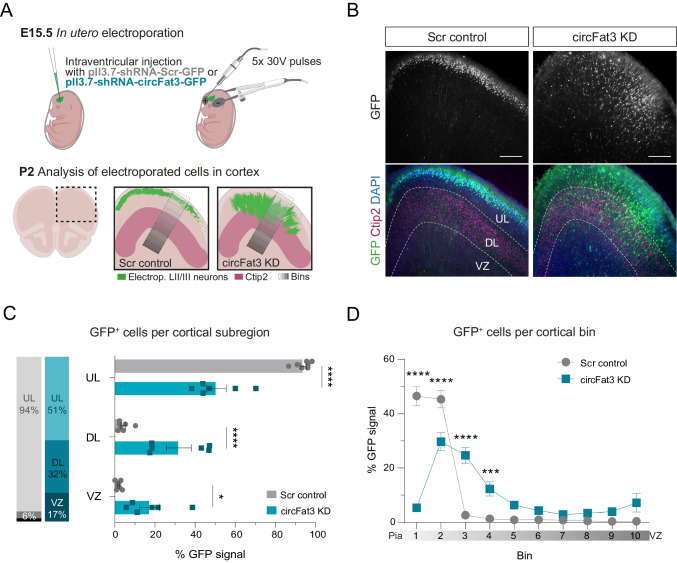
In utero electroporation of murine prefrontal cortex unravels developmental defects for neural progenitors lacking circFat3 expression. **A** Method showing in utero electroporation of prefrontal cortex at E15.5. Intracerebroventricular injection of scr or circFat3 shRNA in combination with FastGreen followed by electroporation with triple electrode (top). Analysis of electroporated neurons was performed at P2 (bottom). Migration defect of circFat3 KD and the binning analysis is schematically depicted. Green = GFP-positive electroporated LII/III neurons, magenta = Ctip2-positive area, grey gradient = binned region for analysis, scr = scrambled, KD = knockdown. Icons adapted from Biorender. **B** Immunohistochemistry of GFP (electroporated LII/III neurons) and Ctip2 (deep cortical layers) after in utero electroporation with scr control (left) or shRNA for circFat3 KD (right). 60 µm thick vibratome sections; dotted line shows region of Ctip2 expression; UL = upper layers, DL = deeper layers, VZ = ventricular zone; scale bar = 200 µm. **C**, **D** Quantification of neuronal migration defect upon circFat3 KD. **C** Analysis of GFP-positive neuron migration using the bins as shown in (**A**), expressed as percentage of total GFP signal per bin. **D** Percentage of GFP signal per cortical subregion for scr control or circFat3 KD. Summarized values (left) and individual values per brain (right) are shown. Subregions were defined using Ctip2 expression: upper layers (UL) = pia to Ctip2-positive area; deep layers (DL) = Ctip2-positive area; ventricular zone (VZ) = below Ctip2-positive area. Values are presented as mean ± SEM. *n* = 7 pups from 2 mothers (scr) and *n* = 6 pups from 3 mothers (circFat3KD). Statistical comparisons were made using two-way ANOVA followed by Sidak’s multiple comparison test; *p < 0.05, ***p < 0.001, ****p < 0.0001

CircFat3 is highly conserved between mouse and human [12], with a sequence identity of 91%. Hence, we turned to a mouse model to test the role for circFat3 in corticogenesis in vivo. Here, we used a mix of two plasmids containing a GFP cassette and two different shRNAs targeting the circFat3 BSJ or a scrambled (scr) control, which resulted in a specific and efficient KD of circFat3 in vitro using transiently transfected P19 cells upon FACS (Figure S7A). Subsequently, we performed IUE of the dorsal prefrontal cortex in E15.5 mice and analyzed the positioning of the GFP-labelled electroporated cells at postnatal day (P) 2 (Fig. 6A). To this end, the region harboring Ctip2-positive deep layer (DL) neurons was used as orientation for migration analysis.

The electroporated cells carrying the scr control plasmid were predominantly found within upper cortical layers (UL) at P2, corresponding to 94% of GFP signal (Fig. 6 B, C). Upon circFat3 depletion, around half of the GFP-positive cells migrated to the UL, whereas around 32% and 17% of the GFP signal was detected within the DL and ventricular zone (VZ), respectively. However, the overall size of the Ctip2-positive area remained unchanged upon circFat3 KD (Figure S7B). The retention of circFat3 deficient neurons in the VZ (bin 8–10) and DL (4–7), was also seen using binning analysis of the cortex from ventricle to pia (Fig. 6A and D). The mis-localization hints at a migration deficit, a defect in progenitor maturation, and/or an increased differentiation speed upon circFat3 depletion.

## Discussion

To study circRNAs within a complex system like the brain is challenging because of their cell type-specific expression, their regulatory roles within different cell types [8–10, 28, 31], and their concurrent expression from linear host genes, which carry regulatory functions themselves [75].

Here, we identified circRNAs differentially regulated during neuronal differentiation, studied the implications of circFAT3 depletion in human COs, and determined transcriptomic changes at single-cell level, thereby accounting for the cell type heterogeneity of COs. We found that depletion of circFAT3 in COs led to the loss of cortical RGCs/NPCs and mature cortical neurons, as well as significant changes in expression patterns of genes related to axon guidance and cell migration. In accordance with these findings, the downregulation of circFat3 in murine embryonic prefrontal cortex resulted in the mispositioning of cells within the neocortex, establishing a functional impact of circFat3 in mammalian nervous system development.

Initially, we found a global upregulation of circRNAs in our early 2D NPC differentiations, in line with previous studies [12, 53]. It has been speculated that the general increase in circRNA expression during neural differentiation could be related to circRNAs accumulating in more mature cells, due to their high stability [28, 76, 77]. However, in COs, circFAT3 declined at late differentiation stages while ciRS-7 continued to increase, suggesting that passive accumulation cannot solely explain changes in circRNA expression over time. Additionally, linear *FAT3* expression remained constant between days 30 and 90. This type of uncoupling between circular and linear RNA from the same gene has previously been seen during neuronal differentiation [10, 12].

The depletion of circFAT3 in 2D NPC differentiations led to no significant gene expression changes. However, when considering genes that showed substantial fold changes, but not passed the FDR cut-off, we observed pathways related to insulin signaling and insulin resistance to be affected. Both pathways work through PI3K-Akt signaling, which plays a vital role in neurodevelopmental processes [78]. It has previously been shown that circBIRC6 and circCORO1C could maintain the pluripotent state of hESCs by interacting with miRNAs [15], which could also apply to circRNAs during the differentiation of hESCs. CircFAT3 contains two predicted target sites for miR-183, which was downregulated upon circFAT3 KD in COs. Thus, circFAT3 could potentially function as a sponge for miR-183 during neural differentiation. In line with this, previous studies suggested a role for miR-183 and miR-182 in cell proliferation [79], neurite outgrowth [80], neuron survival [81] by regulating FOXO expression, PI3K-Akt signaling in general [79, 81], and possibly in insulin signaling by targeting *Irs1* [82]. For both circFAT3 and *FAT3*, an enrichment within synaptoneurosomes was shown previously [12], and it could thus be speculated that circFAT3 could function as a sponge for miR-183 in synaptoneurosomes. However, these changes could likely be secondary effects and further studies are needed to test if the miRNA is present in these sub-cellular compartments and if there is a direct and functionally relevant interaction between circFAT3 and miR-183.

In COs, in addition to telencephalic NPCs, we identified populations of non-telencephalic NPCs (*FOXG1*-negative) with low levels of *PAX6* and high expression of *CXCR4*, which was also seen in a recent study [65, 66]. We found NPCs in circFAT3-depleted COs that were transcriptionally distinct from CTRL non-telencephalic NPC populations with overall decreased *FAT4*, *PLP1*, *PAX6*, and *WNT7B* levels. In general, deregulated genes were mainly involved in axon guidance and Wnt signaling pathways. Interestingly, *FAT4* downregulation has previously been linked to alterations in neural progenitor morphology, disruption of migration, and cortical heterotopia in COs [74]. The study further showed that the genes altered in migratory deficient neurons affected processes like axon guidance, neuronal migration, and patterning [74]. Similarly, *FAT4* KD in murine developing cortex from E14-P7 resulted in neuronal mispositioning [73] comparable to our findings upon circFat3 KD in murine prefrontal cortex. The mis-localization of electroporated cells within the IZ instead of cortical plate when downregulating circFat3 could either indicate a faster differentiation and maturation, a blockage of progenitor maturation, or a migratory deficit of circFat3-depleted cells. Several of the DEGs found between circFAT3 and CTRL NPCs in day 30 COs are implicated in axon guidance, regulation of migration, and neural differentiation [73, 74, 83–86], further strengthening these hypotheses.

A previous study showed that a conserved circRNA, circSLC45A4, can modulate neocortex formation and that depletion of this circRNA in the developing mouse cortex leads to a decrease in basal progenitor cells and an increase in Cajal-Retzius cells [13]. However, as the study was conducted at an earlier timepoint of murine neocortex development than ours, we cannot draw strong conclusions regarding putative overlapping circRNA functions. Still, in both studies, fewer electroporated cells seemed to reach the cortical plate upon circRNA KD.

We showed that neurons in circFAT3 KD COs demonstrated higher levels of *INSM1*, which is responsible for the delamination of apical NPCs [87] and is thus expressed particularly in basal progenitors and nascent neurons [88]. We also observed decreased expression of mature neuronal markers *NEFL* and *NEFM,* important for the radial growth of axons [89]. Based on this, combined with the increased abundance of IPs in circFAT3 KD COs, we hypothesize that deficiency of circFAT3 results in impaired neuronal maturation and accumulation of IPs. Furthermore, we saw a loss of cortical neural progenitors and mature cortical neurons at day 90 and detected an overall loss of mature MAP2-positive neurons, while no changes in the early post-mitotic neuron marker TUJ1 were observed. This could further hint towards a delay of neuronal differentiation and maturation.

While mature neuronal markers decreased in circFAT3-depleted COs at day 90, we saw an increase of GFAP-positive cells, which is a characteristic intermediate filament marker of astrocytes [90]. Thus, circFAT3 depletion may also have an influence on the switch between neurogenesis and gliogenesis based on human in vitro experiments.

In conclusion, we observed striking transcriptomic changes upon circFAT3 depletion when focusing the analysis on individual cell types using scRNA-seq, illustrating the importance of accounting for cell type variation when studying circRNA function. With the precaution that our data is based on a combination of human in vitro experiments and murine in utero electroporation, we speculate that circFAT3 depletion influences the balance between progenitor state, neurogenesis, and gliogenesis leading to a decrease in neuron maturation and an increased abundance in astrocyte-like cells as well as IPs. In addition, the results of both model systems point towards an involvement of circFAT3 in axon guidance or additional migratory mechanisms. However, future studies are needed to elucidate the specific mechanism of action for circFAT3 in neural development.

## Supplementary Information

Below is the link to the electronic supplementary material.

Supplementary file1 (DOCX 61 KB)

Supplementary file2 (PDF 2473 KB)

Supplementary file3 (XLSX 2260 KB)

Supplementary file4 (XLSX 26 KB)

Supplementary file5 (XLSX 20 KB)

Supplementary file6 (XLSX 214 KB)

Supplementary file7 (XLSX 2799 KB)

Supplementary file8 (XLSX 34 KB)

Supplementary file9 (XLSX 57 KB)

## Data Availability

RNAseq and scRNAseq data are available through the Gene Expression Omnibus (GEO) series accession GSE205666. All additional data and materials are available as Online Resource files.
