# Supplementary material for: A Circular RNA Expressed from the FAT3 Locus Regulates Neural Development
Source: Mol Neurobiol. 2023 Feb 25;60(6):3239–60. doi: 10.1007/s12035-023-03253-7 (PMC10122638; doi:10.1007/s12035-023-03253-7)
Supplement: Supplementary file 1 — Supplementary file1 (DOCX 61 KB) [file 12035_2023_3253_MOESM1_ESM.docx]

A circular RNA expressed from the *FAT3* locus
regulates neural development

Sabine Seeler^1,2,+^, Maria Schertz Andersen^1,+^, Tamas Sztanka-Toth^3^, Mateja Rybiczka-Tešulov^4^, Marleen H. van den Munkhof^4^, Chi-Chih Chang^1^, Muyesier Maimaitili^2,5^, Morten Trillingsgaard Venø^1,6^, Thomas Birkballe Hansen^1^, R. Jeroen Pasterkamp^4^, Agnieszka Rybak-Wolf^3^, Mark Denham^2,5^, Nikolaus Rajewsky^3^, Lasse Sommer Kristensen^1,2,#,*^, and Jørgen Kjems^1,#,*^

1- Interdisciplinary Nanoscience Center, Department of Molecular Biology and Genetics, Aarhus University, 8000 Aarhus C, Denmark

2- Department of Biomedicine, The Skou Building, Aarhus University, 8000 Aarhus C, Denmark

3- Berlin Institute for Medical Systems Biology (BIMSB), MDC Berlin-Mitte, 10115 Berlin, Germany

4- Department of Translational Neuroscience, University Medical Center Utrecht Brain Center, 3584 CG Utrecht, Netherlands

5- Danish Research Institute of Translational Neuroscience, Nordic EMBL Partnership for Molecular Medicine, Aarhus University, 8000C Aarhus, Denmark

6- Omiics ApS, 8200 Aarhus N, Denmark

*^+^ These authors contributed equally to this work.*

*^#^ These authors contributed equally to this work.*

*^*^ Correspondence: jk@mbg.au.dk*

**Online Resource 1-Table of contents:**

Supplementary materials and methods

Supplementary tables (Table S1-S4)

Supplementary figure legends (Figure S1-S6)

Supplementary figures (Figure S1-S6)

**Additional files:**

Online Resource 2. DEGs NPCs

Online Resource 3. BLAST off-targets

Online Resource 4. GO/KEGG pathway analysis NPCs

Online Resource 5. RawData miRNAs in NPCs

Online Resource 6. DEGs in organoid clusters

Online Resource 7. GO/KEGG pathway analysis organoid

Online Resource 8. RawData miRNAs organoids

**Supplementary Materials & Methods**

## Neural differentiation of H9 hESCs: Cerebral organoids

H9 human embryonic stem cell (hESC)- derived cerebral organoids (COs) were generated as described previously [1] with modifications [2, 3]. Briefly, after accutase treatment, 5000 cells/well were seeded in a 96-well plate in 100 μl of embryoid body media (EBM): DMEM/F12 (Invitrogen), 20% Knockout replacement serum (KOSR; Invitrogen), 1x Glutamax (Invitrogen), 1x MEM-NEAA (Sigma), 2% ESC FBS (Gibco), 4 ng/ml bFGF (Peprotech), 50 μM Y-27632 ROCK inhibitor (Millipore), 0.1 mM 2-mercaptoethanol (2-ME; Merck). At day two, 100μl of fresh EBM medium was added containing 4 ng/ml bFGF, 50 μM Y-27632 ROCK inhibitor and at day four 50% of the media was replaced with EBM without bFGF and Y-27632 ROCK inhibitor. On day five, the media was exchanged with neural induction media (NIM): DMEM/F12, 1x N2 supplement (Invitrogen), 1x Glutamax (Invitrogen), 1x MEM-NEAA (Sigma), 10 μg/ml heparin solution (Sigma). At day seven or eight, depending on the degree of neuroectoderm induction, the EBs were embedded in Matrigel (Corning) and maintained in NIM until day 13. Then, the media was replaced with improved differentiation media containing 1:1 DMEM/F12 to Neurobasal media (Invitrogen), 1x N2 supplement (Invitrogen), 1x B27 minus vitamin A supplement (Invitrogen), 1x Glutamax (Invitrogen), 1x MEM-NEAA (Sigma), 0.05 mM 2-mercaptoethanol (2-ME;Merck), insulin solution (Sigma), 3 μM CHIR99021 (Tocris) from day 13 until 16. Media changes were performed every other day. After day 20, the COs were transferred to maturation media (1:1 DMEM/F12: Neurobasal media, 1x N2 supplement (Invitrogen), 1x B27 plus vitamin A (Invitrogen), 1x Glutamax (Invitrogen), 1x MEM-NEAA (Sigma), chemically defined lipid concentrate (Gibco), 0.05 mM 2-mercaptoethanol (2-ME;Merck) solution, insulin solution (Sigma), Ascorbic acid (Sigma), HEPES (Gibco)). After day 30 maturation medium was supplemented with 20 ng/ml BDNF (Peprotech), 20 ng/ml GDNF (Peprotech), 0.5 mM dibutyryl cAMP (Tocris), 1% Matrigel (Corning) in a 6-well on an orbital shaker (80 rpm). Media changes were performed every 3-4 days.

## PAGE Northern blot

For agoshRNA analysis, 30 μg extracted RNA was precipitated in ethanol, resuspended in 30 μl loading buffer (8M urea, 20 mM EDTA, 1% xylen, and 1% bromophenolblue) and loaded on a 12% PAGE gel. After running the gel for 1.5 hours at 12W, RNA was transferred to hybond-N+ membranes (GE Healthcare) and UV-crosslinked. The membranes were cut and hybridized with 5’phospho-labelled DNA oligos (**Table S2**) in Church buffer (0.5 M NaPO4, 7% SDS, 1 mM EDTA, 1% BSA, pH 7.5) at 37°C and washed in 2× SSC with 0.1% SDS at room temperature. The membranes were exposed on phosphorimager screens and analyzed using Image Lab (Bio Rad). miR-15b was used as loading control. Cropped images are shown for each band.

## Lentiviral production and transduction

Lentiviral particles were produced in HEK293T cells from agoshRNA vectors as well as the empty vector as control. The Lipofectamine 3000 protocol (Invitrogen) was used following the manufacturing guidelines and using the envelope plasmid pMD2.G and psPAX2 as packaging plasmid. The viral supernatant was harvested at 48 and 72 h post-transfection and concentrated by ultracentrifugation. The titer of the lentiviral supernatant was determined by transduction of HEK293T cells followed by functional analysis.

For lentiviral transduction, 1*10^5^ H9 cells (passage 90-100) were seeded in a vitronectin-coated center-well dish. After 16h, the media was replaced with 2 ml TeSR-E8 medium supplemented with the appropriate amount of lentiviral supernatant. The amount of virus added was calculated using the following formula: X μl = (number of cells*MOI)/titer of the viral supernatant. An MOI of 50 was used for each virus. After 12- 16 h, viral media was replaced by 2 ml fresh E8 media. After 24 h incubation, infected cells were subjected to 1.5 μg/ml of puromycin (Invitrogen) selection for five days.

Fluorescence activated cell soring (FACS)

Single cell suspensions were prepared using 0.05% Trypsin-EDTA (Gibco- Thermo Fisher Scientific), and cells were resuspended in PBS (Gibco- Thermo Fisher Scientific) with 10% FBS (Gibco- Thermo Fisher Scientific). GFP positive cells were sorted using the BigFoot full spectrum cell sorter (Invitrogen) at the FACS Core Facility, Aarhus University, Denmark. The sort was based on side scatter (SSC), forward scatter (FSC), propidium iodide (PI) fluorescence, and GFP fluorescence and a mock control was used to determine the background GFP fluorescence. RNA from GFP positive P19 cells were harvested directly after sorting.

## NanoString analyses

All NanoString experiments were performed using the NanoString nCounter SPRINT (NanoString Technologies, WA, USA) setup. In short, 200 ng total RNA in RNase-free water was mixed with 8 μl reporter probe master mix. To this mixture 2 μl capture probe was added and immediately after the RNA, reporter and capture probes were hybridized for 20 hours at 65°C. After incubation, each sample was diluted by the addition of 17 μl RNase-free water and loaded on the NanoString cartridge. A custom-designed NanoString CodeSet for circRNAs was used. A panel of capture and reporter probes was designed to target regions of 100 nt overlaying the backsplicing junctions (BSJs) of ciRS-7, circRMST, and circFAT3. For normalization, four linear reference genes (*ACTB*, *PUM1*, *SF3A1,* and *B2M*) were also included in the panel. Probes are listed in **Table S4**.

For miRNA profiling using NanoString technology, the human nCounter miRNA expression panel was used. Sample preparation, hybridization, and nCounter run were performed according to the manufacturer’s guidelines.

## QuantSeq 3’ end RNA sequencing: Library preparation

For library preparation, the QuantSeq 3’mRNA-Seq Library Kit (Lexogen, Vienna, Austria) was utilized according to the manufacturer’s instructions at Department of molecular medicine, Aarhus University Hospital, Denmark. Briefly, RNA input of 100 ng for each sample was used and library preparation was performed in two batches of 31 samples each with an internal control in both plates. Oligo-dT primers were hybridized to the 3’end of mRNA transcripts followed by reverse transcription, RNA removal, and second strand synthesis. After library purification using magnetic beads, the library was amplified producing a cDNA library with adapters for Illumina sequencing. High-throughput sequencing of the purified library was performed on the NovaSeq 6000 system (Illumina, CA, USA) at Department of molecular medicine, Aarhus University Hospital, Denmark, with a 100 bp SE sequencing protocol.

## Single cell RNA-sequencing: Library preparation

For scRNA-seq library preparation, earlier publications were followed [3, 4]. In short, after methanol fixation, cells were pelleted at 3000xg for 5 minutes and rehydrated in 1 ml PBS with 0.01% BSA and 1:100 Superasein (Thermo Fisher Scientific) . The cells were then resuspended in 300 μl rehydration buffer, containing PBS-BSA 0.1% plus 1 U/μl RNase Inhibitor (RiboLock; Thermo Fisher Scientific). Cells were filtered through a 40 μm cell strainer, counted using an automated cell counter (Countess; Thermo Fisher Scientific), and diluted to approximately 350 cells/μl in 250 μl rehydration buffer. Barcoded beads (ChemGenes, Macosko- 2011- 10 (V+)) with chilled lysis buffer (6% Ficoll PM-400, 0.2% Sarkosyl, 20 mM EDTA, 200 mM Tris pH 7.5, 50 mM DTT in nuclease-free water) and cells were used for encapsulation into lipid droplets using the Nadia instrument (Dolomite Bio, Hertfordshire, UK) based on manufacturer’s guidelines. The conceptual principle is based on the DropSeq scRNA-seq approach [5]. Library preparation including recovery of beads, reverse transcription, exonuclease treatment, and second strand synthesis procedures were followed as described previously [3, 4]. After incubation with second strand synthesis mix for 60 minutes at 37°C, beads were washed twice with 1 ml TE/0.01% Tween, once with 1 ml TE, and twice with water. cDNA amplification was achieved by dividing beads into 24 PCR reactions with SMART PCR primer [4] and 2x Kapa HiFi Hotstart Ready mix (Roche, Basel, Switzerland) in a total reaction volume of 50 μl using 4+9 cycle settings. After pooling 25 μl of each PCR reaction, the PCR products were cleaned-up using AMPure XP beads (Beckman Coulter). cDNA library quality and quantity were checked on a Bioanalyzer High Sensitivity Chip (Agilent) and the Qubit dsDNA HS Assay system (ThermoFisher). For each sample, 1000 pg of the cDNA library was used for Tagmenation using Nextera XT v2 DNA sample preparation (Illumina) including custom primers for amplification purposes. After PCR purification, quality and quantity assessment, paired-end sequencing was performed on a NextSeq500 (Illumina) and the NextSeq 500/550 High Output v2 Kit (75 cycles) (Read1CustSeqB custom primer for read 1 = 21 bp; read 2 = 63 bp) [3, 4].

## BaseScope single molecule RNA FISH

*In situ* hybridization (ISH) was performed using BaseScope technology (ACD, Newark, CA, USA) following the instructions provided by the manufacturer (ACD#323900). In short, CO tissue sections were incubated in warm PBS for 15 minutes to remove embedding media, incubated for 30 minutes at 60°C using the HybEZ II hybridization oven (ACD), and post-fixed with 4% PFA for 15 minutes. After following the dehydration series, sections were air dried and incubated with Hydrogen Peroxide (ACD) for 10 minutes at room temperature. After two washes with water (ddH_2_O) and rinsing in 100% ethanol, the sections were air dried overnight. After protease plus treatment for 15-30 minutes at 40°C, two custom hybridization probes covering the BSJ of circFAT3 or ciRS-7 were applied, and sections were incubated at 40°C for 2 hours. The subsequent amplification steps and application of the Fast RED solution (ACD) were performed according to the provider’s protocol. Sections were counterstained with DAPI (Thermo Fisher Scientific, 1 μg/ml) for 10 minutes, washed, and mounted using ProLong Gold antifade (Invitrogen). Image acquisition was achieved with Zeiss laser scanning confocal LSM800 or the Olympus VS120 Slide Scanner System.

## Marker quantification in COs

For MAP2, TUJ1, and GFAP quantification, 90-day COs from two batches with three to four COs per batch were analyzed using Fiji [6]. Due to the lack of clear ventricle-like structures at day 90, we defined six regions of interest (ROI) per organoid section each with a dimension of 250 x 250 μm at the most basal position of the cortical plate. In total we analyzed three non-consecutive sections per CO for quantification. For image processing, we applied similar manual thresholding within batches, gaussian blur, and created a binary image. The signal intensity measurements were averaged across 18 ROI’s for each CO and normalized to the area analyzed (μm^2^). For statistical analysis, a two-tailed unpaired t-test was used.

## Quantification of migration defect of *in utero* electroporated brains

After immunohistochemistry, brain sections were imaged on a Zeiss Axiovision epifluorescent microscope with a 10x objective. Per brain, three images of the prefrontal cortex from consecutive sections were used for quantifications in Fiji [6]. Migration of GFP-expressing cells was quantified by manual thresholding, followed by applying the ‘Gaussian blur’ and ‘binarization’ plug-ins. Then, ten equal bins with 250 μm (width) by 145 μm (height) dimensions were delineated from the pia to the ventricular zone. For each bin, the GFP signal intensity was measured and compared to the total GFP signal intensity of all bins to obtain percentage values. Subsequently, within the binned area we manually marked the Ctip2-positive region as well as the Ctip2-negative regions above (upper layers) and below (ventricular zone) as ROI’s. We then measured the GFP signal intensity in the three separate ROI’s and compared these absolute values to the total GFP signal intensity to obtain percentage values. We also measured the area of the Ctip2-positive ROI. Mean values of the three images per brain were used for statistical analysis. Statistical comparisons were performed using two-way ANOVA followed by Sidak’s multiple comparisons for the percentage of GFP signal per cortical bin and per cortical subregion, and a Mann Whitney-U test for size of the Ctip2-positive area.

## NanoString data analyses

The raw data for the custom-designed panel was processed using the nSOLVER 4.0 software (NanoString Technologies, WA, USA). First, a background subtraction was performed using the max of negative controls, followed by the normalization to a positive control using the geometric mean of all positive controls. Finally, a second normalization to the geometric mean of the three most stable linear reference genes (*GAPDH*, *PUM1,* and *SF3A1*) was performed, before exporting the data.

For the analysis of the miRNA panel, the R package NanoStringDiff [7] was used to perform background subtraction, normalization to negative controls as well as housekeeping genes, and differential gene expression analysis. Additionally, the algorithm NormFinder [8] was used to identify the optimal housekeeping genes among the set of candidates for normalization. Here the genes *ACTB*, *GAPDH*, *RPL19*, and *RPLP0* were used for normalization.

References

1. Lancaster MA, Corsini NS, Wolfinger S, Gustafson EH, Phillips AW, Burkard TR, Otani T, Livesey FJ, Knoblich JA (2017) Guided self-organization and cortical plate formation in human brain organoids. Nat Biotechnol 35:659–666. https://doi.org/10.1038/nbt.3906

2. Quadrato G, Nguyen T, Macosko EZ, Sherwood JL, Min Yang S, Berger DR, Maria N, Scholvin J, Goldman M, Kinney JP, Boyden ES, Lichtman JW, Williams ZM, McCarroll SA, Arlotta P (2017) Cell diversity and network dynamics in photosensitive human brain organoids. Nature 545:48–53. https://doi.org/10.1038/nature22047

3. Rybak-Wolf A, Wyler E, Legnini I, Loewa A, Glažar P, Kim SJ, Pentimalli TM, Martinez AO, Beyersdorf B, Woehler A, Landthaler M, Rajewsky N (2021) Neurodegeneration in human brain organoids infected with herpes simplex virus type 1. bioRxiv 2021.03.05.434122. https://doi.org/10.1101/2021.03.05.434122

4. Emanuel W, Kirstin M, Vedran F, Asija D, Theresa GL, Roberto A, Filippos K, David K, Katja H, Salah A, Christopher B, Karen H, Anja R, Ivano L, Andranik I, Tommaso M, Simone DG, Jan P, Samantha P, Meyer Thomas F, Alexander MM, Daniela N, Andreas H, Matthias S, Altuna A, Nikolaus R, Christian D, Markus L (2021) Transcriptomic profiling of SARS-CoV-2 infected human cell lines identifies HSP90 as target for COVID-19 therapy. iScience 24:102151. https://doi.org/10.1016/j.isci.2021.102151

5. Macosko EZ, Basu A, Satija R, Nemesh J, Shekhar K, Goldman M, Tirosh I, Bialas AR, Kamitaki N, Martersteck EM, Trombetta JJ, Weitz DA, Sanes JR, Shalek AK, Regev A, McCarroll SA (2015) Highly parallel genome-wide expression profiling of individual cells using nanoliter droplets. Cell 161:1202–1214. https://doi.org/10.1016/j.cell.2015.05.002

6. Schindelin J, Arganda-Carreras I, Frise E, Kaynig V, Longair M, Pietzsch T, Preibisch S, Rueden C, Saalfeld S, Schmid B, Tinevez J-Y, White DJ, Hartenstein V, Eliceiri K, Tomancak P, Cardona A (2012) Fiji: an open-source platform for biological-image analysis. Nat Methods 9:676–682. https://doi.org/10.1038/nmeth.2019

7. Wang H, Horbinski C, Wu H, Liu Y, Sheng S, Liu J, Weiss H, Stromberg AJ, Wang C (2016) NanoStringDiff: A novel statistical method for differential expression analysis based on NanoString nCounter data. Nucleic Acids Res 44:e151. https://doi.org/10.1093/nar/gkw677

8. Andersen CL, Jensen JL, Ørntoft TF (2004) Normalization of real-time quantitative reverse transcription-PCR data: A model-based variance estimation approach to identify genes suited for normalization, applied to bladder and colon cancer data sets. Cancer Res 64:5245–5250. https://doi.org/10.1158/0008-5472.CAN-04-0496

9. Dudekula DB, Panda AC, Grammatikakis I, De S, Abdelmohsen K, Gorospe M (2016) CircInteractome: A web tool for exploring circular RNAs and their interacting proteins and microRNAs. RNA Biol 13:34–42. https://doi.org/10.1080/15476286.2015.1128065

**Supplementary Tables**

**Table S1. DNA oligos agoshRNAs**

| AgoshRNA_circRMST_2_fw | caccAATCCTGAGAATCTCATGAAGCCATGAGATTCTCAGGAT |
| --- | --- |
| AgoshRNA_circRMST_2_re | aaagATCCTGAGAATCTCATGGCTTCATGAGATTCTCAGGATT |
| AgoshRNA_circFAT3_2_fw | caccAATACTTCCATCCACTCTCGTCAGAGTGGATGGAAGTAT |
| AgoshRNA_circFAT3_2_re | aaagATACTTCCATCCACTCTGACGAGAGTGGATGGAAGTATT |
| AgoshRNA_ciRS-7_2_fw | caccACATCGGAAACCCTGGATATTGATCCAGGGTTTCCGATG |
| AgoshRNA_ciRS-7_2_re | aaagCATCGGAAACCCTGGATCAATATCCAGGGTTTCCGATGT |
| sh_circFat3_1_fw | TAGATGATGAGAGTGGACGGACTTGGATCCAAGTCCGTCCACTCTCATCATCTTTTTTCC |
| sh_circFat3_1_re | TCGAGGAAAAAAGATGATGAGAGTGGACGGACTTGGATCCAAGTCCGTCCACTCTCATCATCTA |
| sh_circFat3_2_fw | TTGAGAGTGGACGGACATGTGATTGGATCCAATCACATGTCCGTCCACTCTCATTTTTCC |
| sh_circFat3_2_re | TCGAGGAAAAATGAGAGTGGACGGACATGTGATTGGATCCAATCACATGTCCGTCCACTCTCAA |

**Table S2. Northern blot oligos**

| circRMST_agoshRNA | GCTTCATGAGATTCTCAGGAT |
| --- | --- |
| circFAT3_agoshRNA | GACGAGAGTGGATGGAAGTAT |
| ciRS-7_agoshRNA | CAATATCCAGGGTTTCCGATG |
| miR-15b | TGTAAACCATGATGTGCTGCTA |

**Table S3. RT-qPCR primers**

| **Primers (human):** |  |  |  |
| --- | --- | --- | --- |
| FAT3 FW | ATCAGGGATGGCAGTGGTCTT | GFAP FW | TCCTGGAACAGCAAAACAAG |
| circFAT3 RE | TGCCCACACAGTGTCCCATA | GFAP RE | CAGCCTCAGGTTGGTTTCAT |
| linearFAT3 RE | ATTGTCTCCCGATCAAGAATGTC | SOX2 FW | ACCAGCGCATGGACAGTTAC |
| ciRS-7 FW | CTTGACACAGGTGCCATC | SOX2 RE | CCGTTCATGTAGGTCTGCGA |
| ciRS-7 RE | ACGTCTCCAGTGTGCTGA | TTR FW | TGGAAGGCACTTGGCATCTC |
| RMST RE | AGGGGCTAGTTGAGGAATGG | TTR RE | TCCTTGGGATTGGTGACGAC |
| circRMST FW | GCAGACTTCTTTGGGCATGT | S100b FW | AGGGAGGGAGACAAGCACAA |
| linearRMST FW | ATGAGAAGTGACATCCTCCTC | S100b RE | ACTCGTGGCAGGCAGTAGTA |
| GAPDH FW | GTCAGCCGCATCTTCTTTTG | SULF1 FW | CACACGGTAGAACGAGGCAT |
| GAPDH RE | GCGCCCAATACGACCAAATC | SULF1 RE | CTGTGTAGGTCATAGCTTCCTCC |
| SF3A1 FW | TCCATCCGTGAGAAGCAGAGC | WNT7B FW | TCCCTGGATCATGCACAGAAAC |
| SF3A1 RE | TCTGGATCTCCTCCTCACCG | WNT7B RE | GGATGACAGTGCTCCGAGCTT |
| PAX6 FW | AACAGACACAGCCCTCACAA | FAT4 FW | CAGGAATTCCATCGCCTCCA |
| PAX6 RE | TCATAACTCCGCCCATTCACC | FAT4 RE | TCCTGGCTCCCCGCA |
| SLC17A7 FW | CATGAACCCCCTCACGAAGT | SYP FW | TGGGGACTACTCCTCGTCAG |
| SLC17A7 RE | TGAGCAGCAGGTAGAACGTC | SYP RE | GTGGCCAGAAAGTCCAGCAT |
| NES FW | CGCACCTCAAGATGTCCCTC | UNC5C FW | TCATAAAGCAGGCCCGACTC |
| NES RE | CAGCTTGGGGTCCTGAAAGC | UNC5C RE | ACCAGCCACCGTTGACATAG |
| DCX FW | ACTCAGCAAACGGAACCTCC | EPHA3 FW | TGATTCCGCAGCCTTCCAAT |
| DCX RE | AGGTACAGGTCCTTGTGCTTC | EPHA3 RE | CTGATCTCTTCCCACCCATGT |
| **Primers (mouse):** |  |  |  |
| circFat3 FW | TGAGGATTCTGGTAGGGATGGA | Rpl13a FW | GGGGTTGGTATTCATCCGCT |
| circFat3 RE | AAGAAGGAGGCTTTGTGCCC | Rpl13a RE | TGTGGCCAAGCAGGTACTTC |
| linear Fat3 FW | GTTTGACATCGTCGGGGG | Gapdh FW | GGTGAAGGTCGGTGTGAACG |
| linear Fat3 RE | TTGTTCCATCGGTGACCTCG | Gapdh RE | CTCGCTCCTGGAAGATGGTG |

**Table S4. Nanostring Probe target region**

| **Class** | **NAME (position_sense gene_antisense gene** | **PROBE SEQUENCE** |
| --- | --- | --- |
| circRNA | chr12:97886238-97954825_circRMST_. | GAATAATTCCAGCAAGTGCCAAGGGGCTAGTTGAGGAATGGCTTCATGAGATTCTCAGGATGATGGAGTGAGTGATGGAATAGGTTGCCAACTGTAGTTA |
| circRNA | chrX:139865339-139866824_.ciRS-7 | AACGTCTCCAGTGTGCTGATCTTCTGACATTCAGGTCTTCCAGTGTCTGCAATATCCAGGGTTTCCGATGGCACCTGTGTCAAGGTCTTCCAACAACTCC |
| circRNA | chr11:92085261-92088570_circFAT3_. | CATCAGGGATGGCAGTGGTCTTGGAAGGTTCAGTATAGACGACGAGAGTGGATGGAAGTATGATGTGATGGATATAATTATGGGACACTGTGTGGGCACA |
| lncRNA | linear RMST | TTTCATTTGTGATTCGGATGATGCAGCTCTAGGTGGATTGACTATGAAAGGCGCTGAATATCTTCAGGAAAATGGGTTCCATGAGAAGTGACATCCTCCT |
| mRNA | GAPDH | GGTCTCCTCTGACTTCAACAGCGACACCCACTCCTCCACCTTTGACGCTGGGGCTGGCATTGCCCTCAACGACCACTTTGTCAAGCTCATTTCCTGGTAT |
| mRNA | ACTB | TGCAGAAGGAGATCACTGCCCTGGCACCCAGCACAATGAAGATCAAGATCATTGCTCCTCCTGAGCGCAAGTACTCCGTGTGGATCGGCGGCTCCATCCT |
| mRNA | PUM1 | CTGGGGAACATCAGATCATTCAGTTTCCCAGCCAATCATGGTGCAGAGAAGACCTGGTCAGAGTTTCCATGTGAACAGTGAGGTCAATTCTGTACTGTCC |
| mRNA | SF3A1 | CTTCTAAGCCAGTTGTGGGGATTATTTACCCTCCTCCAGAGGTCAGAAATATTGTTGACAAGACTGCCAGCTTTGTGGCCAGAAACGGGCCTGAATTTGA |
| mRNA | B2M | GTCTGGGTTTCATCCATCCGACATTGAAGTTGACTTACTGAAGAATGGAGAGAGAATTGAAAAAGTGGAGCATTCAGACTTGTCTTTCAGCAAGGACTGG |

**Supplementary figure legends**

**Figure S1 Global increase of circRNA expression upon differentiation of human embryonic stem cells into rostral and caudal neural progenitors**

A) Number of unique circRNAs detected in H9 hESCs, NEPs, NMPs, rNPCs, and cNPCs supported by at least 75 BSJ reads using RNA-sequencing; n=3. Number of junction-spanning reads for all detected circRNAs is shown and circRNA candidates are indicated.

B) Principal component analysis (PCA) plot of H9 hESCs, NEPs, NMPs, rNPCs, and cNPCs based on expression data for the 417 high-abundance circRNAs; n=3.

C) Number of reads spanning the BSJ of circFAT3, ciRS-7, and circRMST at day 0, 4, and 11 of differentiation into rNPCs (left panel) and cNPCs (right panel). Mean values per time point ± SEM are shown; n=3.

D) Number of reads spanning the splice donor- and splice acceptor sites of the same BSJ as the cognate circRNA for linear *FAT3* and linear *RMST* at day 0, 4, and 11 of differentiation into rNPCs (left panel) and cNPCs (right panel). Mean values per time point ± SEM are shown; n=3.

BSJ: backsplicing junction, hESC: human embryonic stem cells, NEPs: neuroepithelial progenitor cells, NMPs: neuromesodermal progenitor cells, rNPCs/cNPCs: rostral/caudal neural progenitor cells, DIV: days in vitro.

## **Figure S2 Depletion of circFAT3 leads to minor gene expression changes in early neural differentiation**

A) Left panel: predicted secondary structure of a folded agoshRNA. AGO2 cut site is marked by black arrowhead. The mature agoshRNA sequence is marked in green. Right panel: location of mature agoshRNA sequence on BSJ of circRNA.

B) Northern blot of small RNAs detected using probes targeting the mature guide strand RNAs for circRMST, circFAT3, and ciRS-7. A hsa-miR-15b probe was used as loading control.

C)-D) Assessment of circRNA KD for ciRS-7, circRMST, and circFAT3 with RT-qPCR in cNPCs (C) and rNPCs (D). Expression was normalized to *GAPDH* expression. Plots show mean fold change (FC) compared to CTRL ± SEM; n=3; one-sample t-test on log2-transformed values; *p<0.05, **p<0.01, ***p<0.001.

E) Quantification of linear *FAT3* in circFAT3 KD and CTRL cNPCs (left panel) and rNPCs (right panel) using RT-qPCR. Expression levels were normalized to the mean of *GAPDH* and *SF3A1*; n=3. Mean ± SEM are shown; two-tailed unpaired t-tests.

F) Quantification of linear *RMST*, circRMST KD, and CTRL cNPCs using NanoString nCounter technology, shown as normalized counts; n=3. Mean ± SEM are shown; two-tailed unpaired t-tests.

G) Quantification of *PRDX6* expression in circRMST KD and CTRL SH-SY5Y cells using RT-qPCR. Expression levels were normalized to *GAPDH*. Mean or mean ± SEM are shown for n≤2 or n>2, respectively. N (DIV 0)= 2; n (DIV 8)= 3.

H) Venn diagram showing overlap of DEGs between ciRS-7 KD and circFAT3 KD in hESCs, rNPCs, and cNPCs.

BSJ: backsplicing junction, hESC: human embryonic stem cells, NEPs: neuroepithelial progenitor cells, NMPs: neuromesodermal progenitor cells, rNPCs/cNPCs: rostral/caudal neural progenitor cells, DIV: days in vitro.

**Figure S3 Depletion of ciRS-7 and circFAT3 leads to minor miRNA changes in early neural differentiation**

A) Barplot showing predicted number of miRNA binding sites for circFAT3 (top) and ciRS-7 (bottom) based on CircInteractome predictions [9]. Plotted are only miRNAs that showed more than two predicted binding sites for the respective circRNA.

B) Clustered heatmap of differentially expressed (DE) miRNAs in rNPCs and cNPCs detected using NanoString technology. Normalized counts were log2-transformed (normalized counts + pseudocount) and converted to a z-scale; n=3.

C)-F) MA plots of DE miRNA changes in ciRS-7-deficient rNPCs at day 11 (C), circFAT3-deficient rNPCs at day 11 (D), ciRS-7-deficient cNPCs at day 11 (E), and circFAT3-deficient cNPCs at day 11 (F); n=3.

BSJ: backsplicing junction, hESC: human embryonic stem cells, rNPCs/cNPCs: rostral/caudal neural progenitor cells, DIV: days in vitro.

**Figure S4 Establishment of cerebral organoids (COs) to study circRNA function during later stages of human brain development**

A) Quantification of cell numbers per cluster derived from either of the two CTRL replicates in day 90 COs. Clusters defined as indicated in Figure 3E.

B) Heatmap of selected genes (depicted as mean z-score across cells within a cluster) characterizing the day 90 CTRL organoid clusters identified in Figure 3E.

C) Immunohistochemical (IHC) images of TUJ1 (grey) and GFAP (red)-positive cells in day 90 CTRL organoids. 12 μm cryostat sections; scale bar = 50 μm.

D) IHC images of day 90 CTRL COs. SOX2-positive cells surrounding the ventricle-like structure. MAP2= yellow; SOX2= red; DAPI= blue. 12 μm cryostat sections; scale bar = 50 μm.

E-F) Subcellular localization of ciRS-7 (E) and circFAT3 (F) using ISH of COs sections at day 30. Cell nuclei were stained with DAPI (blue) and cell cytoplasm were GFP^+^ (green) through agoshRNA expression vector. White arrowheads indicate subcellular localization of ciRS-7. 12 μm cryostat sections; scale bar = 15 μm.

**Figure S5 Depletion of circFAT3 alters abundance of forebrain radial glial cells in day 30 cerebral organoids (COs)**

A) CircRNA ISH using BaseScope technology in CTRL (top) and circRNA KD (bottom) day 30 COs. Right image (merge): ciRS-7 and circFAT3 (pink), DAPI (blue), and GFP^+^ (green) by agoshRNA expression vector. Left image (circRNA only): ciRS-7 and circFAT3 (grey). 12 μm cryostat sections; scale bar = 20 μm.

B) Quantification of ciRS-7 KD using RT-qPCR. For day 30 and 90 COs, KD efficiency was 69.0% ± 9.9% and 87.7% ± 3.6%, respectively. Expression was normalized to the mean of *GAPDH* and *SF3A1*. Plots show mean ± SEM; n=4 from two CO batches; two-tailed unpaired t-test, **p<0.01, ***p<0.001.

C) Quantification of circFAT3 KD on linear *FAT3* expression using RT-qPCR. For day 30 and 90 COs a downregulation of 6.8% ± 12.6% and -2.4% ± 8.9% was determined, respectively. Expression was normalized to the mean of *GAPDH* and *SF3A1*. Plots show mean ± SEM; n=4 from two CO batches; two-tailed unpaired t-test.

D) Validation of DEGs determined between single cell clusters of non-telencephalic neural progenitor cells (NPCs; Figure 4G) in day 30 CTRL and circFAT3-deficient COs using RT-qPCR on bulk RNA. Expression was normalized to the mean of *GAPDH* and *SF3A1*. Plots show mean fold change (FC) ± SEM compared to CTRL; n=4 from two CO batches; one-sample t-test on log2-transformed values; *p<0.05.

E) Gene Ontology (GO) analysis of DEGs (p.adj<0.05; |log2FC| >1) identified between circFAT3 KD- and CTRL-specific non-telencephalic NPCs. Depicted are GO terms related to biological processes (BP).

F) MA plot showing DEGs identified between circFAT3 KD- and CTRL-specific Neurons II using DESeq2. Mean expression is plotted against log2 fold changes.

**Figure S6 Depletion of circFAT3 leads to loss of neuronal progenitor- and mature cortical neuron-populations in day 90 telencephalic cerebral organoids (COs)**

A)-B) Validation of down-(A) and upregulated (B) markers from single cell transcriptomics in day 90 COs using RT-qPCR on bulk RNA. Expression was normalized to *GAPDH* and *SF3A1*. Plots show mean fold change (FC) ± SEM compared to CTRL; n=4 from two CO batches; one-sample t-test on log2-transformed values; *p<0.05, **p<0.01, ***p<0.001.

C) Log-normalized expression of selected marker genes from Figure 5A and B, showing per-cluster specificity and abundance per cell for each gene.

D) Quantification of DAPI and SOX2 signals in day 90 CTRL and circFAT3 COs. The signal intensity was normalized to the area analyzed (μm^2^). Values are presented as mean ± SEM. N=6 (CTRL) and n=7 (circFAT3) from two CO batches. Two-tailed unpaired t-test, ns: not significant.

E) IHC images of day 90 circFAT3-depleted COs. White arrows indicate exclusive expression of either GFAP or TUJ1. GFAP= red; TUJ1= grey; DAPI= blue. 12 μm cryostat sections; scale bar = 20 μm.

**Figure S7 *In utero* electroporation of murine prefrontal cortex unravels developmental defects for neural progenitors lacking circFat3 expression**

A) Validation of circFat3 knockdown efficiency of two shRNAs using RT-qPCR in P19 cells after transfection and subsequent fluorescence activated cell sorting (FACS) of GFP positive cells. Values are presented as mean ± SEM. n=2 (Scr) and n=3 (circFat3 KD I and II). Two-way ANOVA with correction for multiple testing; ****p<0.0001, ns = not significant.

B) Size of Ctip2+ area in scr control and circFat3 KD brain sections. Values are presented as mean ± SEM. n=7 pups from 2 mothers (Scr) and n=6 pups from 3 mothers (circFat3 KD). Mann-Whitney test (MWU = 20, p = 0.945); ns = not significant.
