# Supplementary material for: A Circular RNA Expressed from the FAT3 Locus Regulates Neural Development
Source: Mol Neurobiol. 2023 Feb 25;60(6):3239–60. doi: 10.1007/s12035-023-03253-7 (PMC10122638; doi:10.1007/s12035-023-03253-7)
Supplement: Supplementary file 2 — Supplementary file2 (PDF 2473 KB) [file 12035_2023_3253_MOESM2_ESM.pdf]

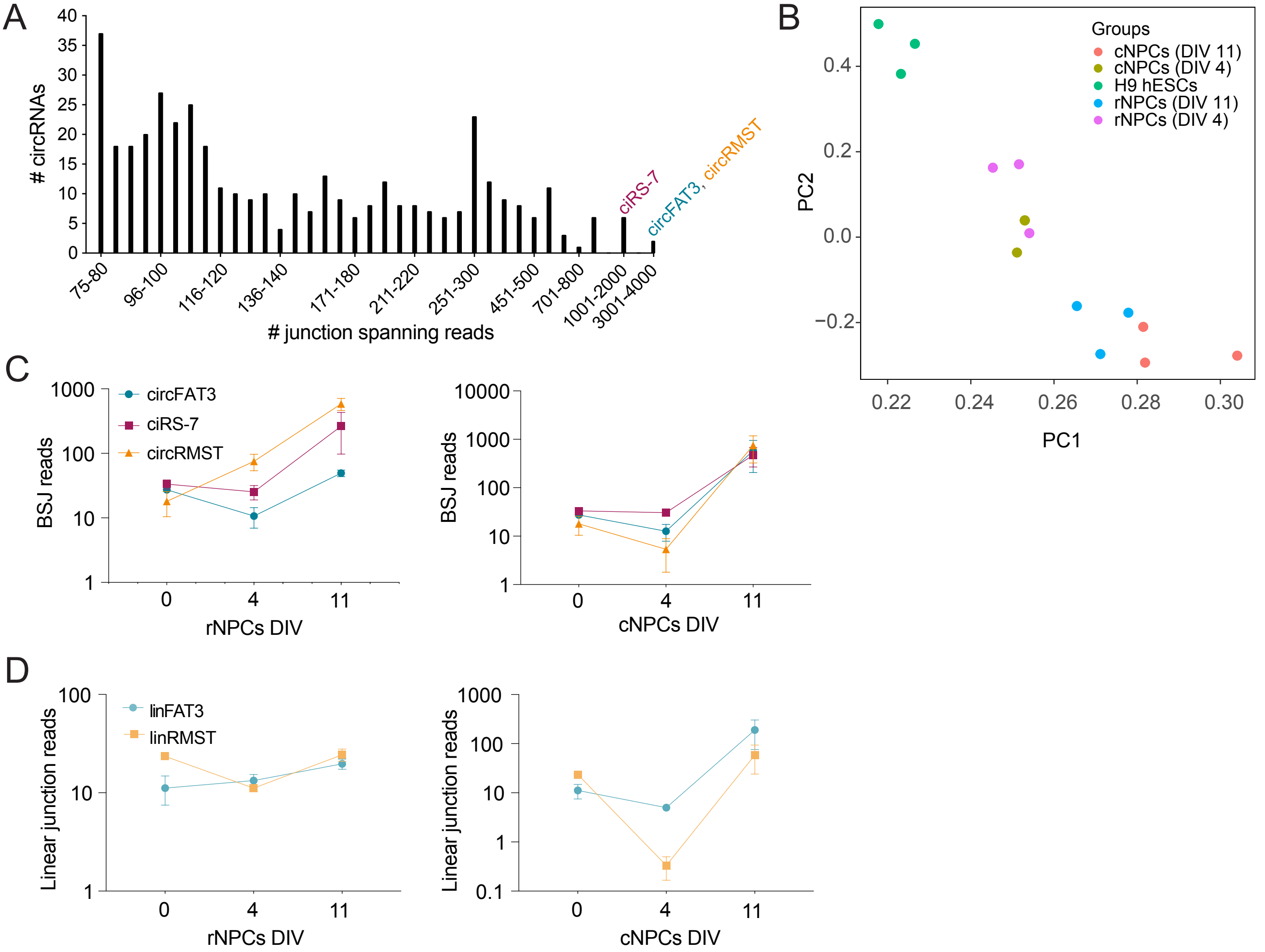
A
circRNAs
junction spanning reads
75-80
96-100
116-120
136-140
171-180
211-220
251-300
451-500
701-800
1001-2000
3001-4000
ciRS-7
circFAT3, circRMST
B
Groups
cNPCs (DIV 11)
cNPCs (DIV 4)
H9 hESCs
rNPCs (DIV 11)
rNPCs (DIV 4)
PC1
PC2
C
circFAT3
ciRS-7
circRMST
BSJ reads
rNPCs DIV
cNPCs DIV
D
linFAT3
linRMST
Linear junction reads
rNPCs DIV
cNPCs DIV

Figure S1

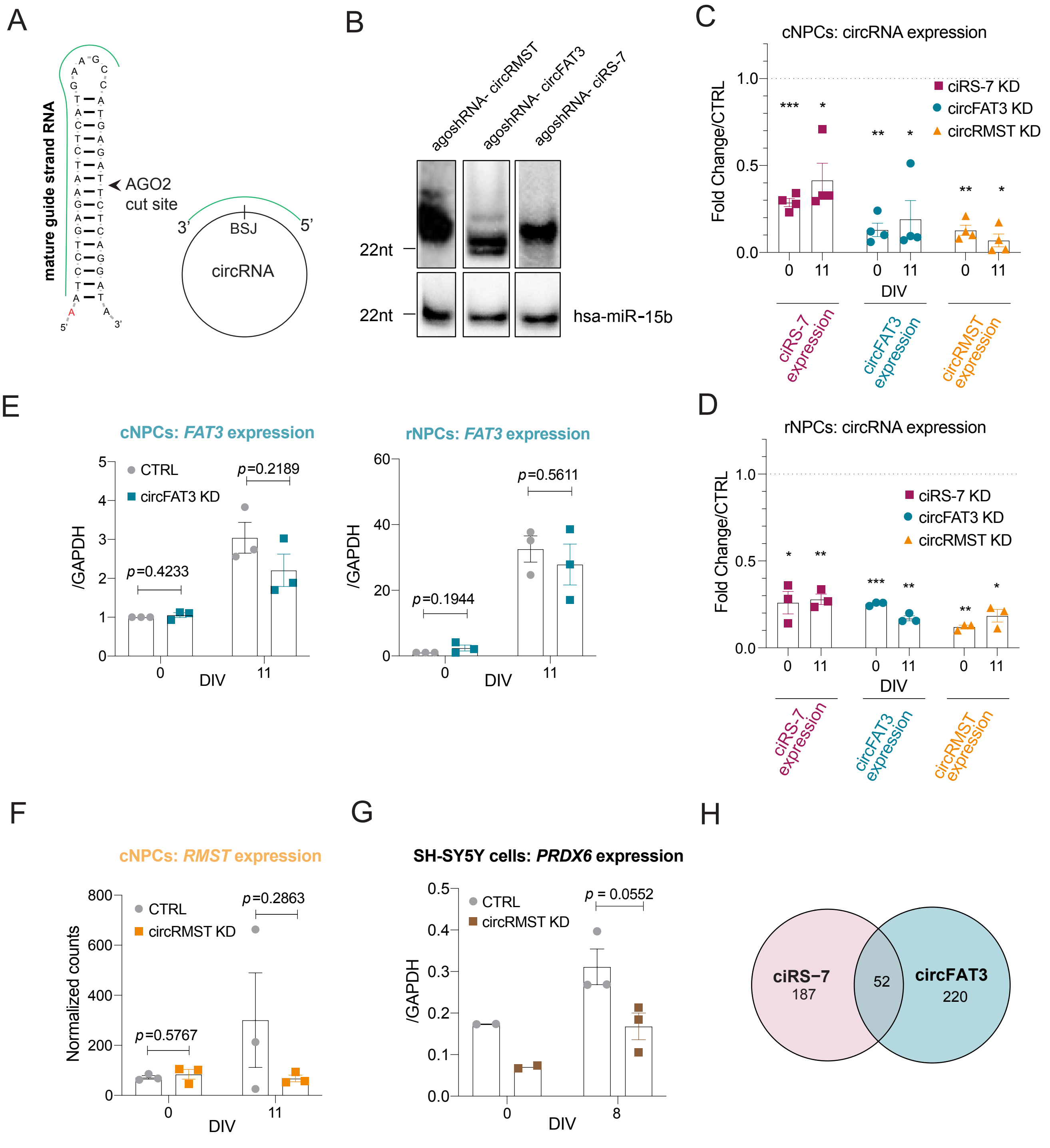
A
mature guide strand RNA
AGO2 cut site
BSJ
circRNA
3'
5'
B
agoshRNA- circRMST
agoshRNA- circFAT3
agoshRNA- ciRS-7
22nt
22nt
hsa-miR-15b
C
cNPCs: circRNA expression
Fold Change/CTRL
ciRS-7 KD
circFAT3 KD
circRMST KD
DIV
ciRS-7 expression
circFAT3 expression
circRMST expression
D
rNPCs: circRNA expression
Fold Change/CTRL
ciRS-7 KD
circFAT3 KD
circRMST KD
DIV
ciRS-7 expression
circFAT3 expression
circRMST expression
E
cNPCs: FAT3 expression
CTRL
circFAT3 KD
p=0.4233
p=0.2189
/GAPDH
DIV
rNPCs: FAT3 expression
p=0.1944
p=0.5611
/GAPDH
DIV
F
cNPCs: RMST expression
CTRL
circRMST KD
p=0.5767
p=0.2863
Normalized counts
DIV
G
SH-SY5Y cells: PRDX6 expression
CTRL
circRMST KD
p = 0.0552
/GAPDH
DIV
H
ciRS−7
187
52
circFAT3
220

Figure S2

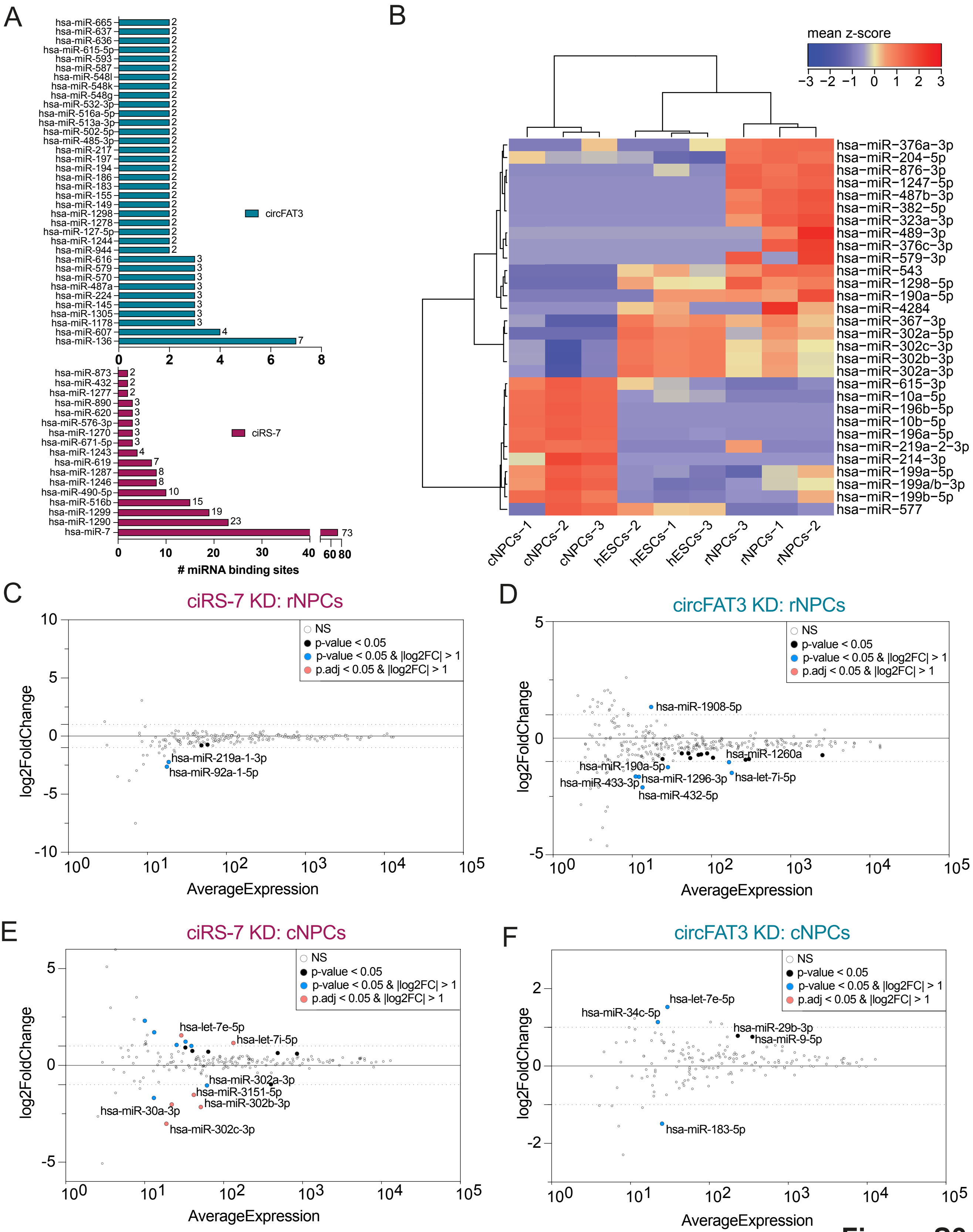

A
hsa-miR-665 2
hsa-miR-637 2
hsa-miR-636 2
hsa-miR-615-5p 2
hsa-miR-593 2
hsa-miR-587 2
hsa-miR-548l 2
hsa-miR-548k 2
hsa-miR-548g 2
hsa-miR-532-3p 2
hsa-miR-516a-5p 2
hsa-miR-513a-3p 2
hsa-miR-502-5p 2
hsa-miR-485-3p 2
hsa-miR-217 2
hsa-miR-197 2
hsa-miR-194 2
hsa-miR-186 2
hsa-miR-183 2
hsa-miR-155 2
hsa-miR-149 2
hsa-miR-1298 2
hsa-miR-1278 2
hsa-miR-127-5p 2
hsa-miR-1244 2
hsa-miR-944 2
hsa-miR-616 3
hsa-miR-579 3
hsa-miR-570 3
hsa-miR-487a 3
hsa-miR-224 3
hsa-miR-145 3
hsa-miR-1305 3
hsa-miR-1178 3
hsa-miR-607 4
hsa-miR-136 7
circFAT3
hsa-miR-873 2
hsa-miR-432 2
hsa-miR-1277 2
hsa-miR-890 3
hsa-miR-620 3
hsa-miR-576-3p 3
hsa-miR-1270 3
hsa-miR-671-5p 3
hsa-miR-1243 4
hsa-miR-619 7
hsa-miR-1287 8
hsa-miR-1246 8
hsa-miR-490-5p 10
hsa-miR-516b 15
hsa-miR-1299 19
hsa-miR-1290 23
hsa-miR-7 73
ciRS-7
miRNA binding sites
B
mean z-score
hsa−miR−376a−3p
hsa−miR−204−5p
hsa−miR−876−3p
hsa−miR−1247−5p
hsa−miR−487b−3p
hsa−miR−382−5p
hsa−miR−323a−3p
hsa−miR−489−3p
hsa−miR−376c−3p
hsa−miR−579−3p
hsa−miR−543
hsa−miR−1298−5p
hsa−miR−190a−5p
hsa−miR−4284
hsa−miR−367−3p
hsa−miR−302a−5p
hsa−miR−302c−3p
hsa−miR−302b−3p
hsa−miR−302a−3p
hsa−miR−615−3p
hsa−miR−10a−5p
hsa−miR−196b−5p
hsa−miR−10b−5p
hsa−miR−196a−5p
hsa−miR−219a−2−3p
hsa−miR−214−3p
hsa−miR−199a−5p
hsa−miR−199a/b−3p
hsa−miR−199b−5p
hsa−miR−577
cNPCs−1 cNPCs−2 cNPCs−3 hESCs−2 hESCs−1 hESCs−3 rNPCs−3 rNPCs−1 rNPCs−2
C
ciRS-7 KD: rNPCs
NS
p-value < 0.05
p-value < 0.05 & |log2FC| > 1
p.adj < 0.05 & |log2FC| > 1
hsa-miR-219a-1-3p
hsa-miR-92a-1-5p
log2FoldChange
AverageExpression
D
circFAT3 KD: rNPCs
hsa-miR-1908-5p
hsa-miR-1260a
hsa-miR-190a-5p
hsa-miR-433-3p
hsa-miR-1296-3p
hsa-let-7i-5p
hsa-miR-432-5p
E
ciRS-7 KD: cNPCs
hsa-let-7e-5p
hsa-let-7i-5p
hsa-miR-302a-3p
hsa-miR-3151-5p
hsa-miR-302b-3p
hsa-miR-30a-3p
hsa-miR-302c-3p
F
circFAT3 KD: cNPCs
hsa-let-7e-5p
hsa-miR-34c-5p
hsa-miR-29b-3p
hsa-miR-9-5p
hsa-miR-183-5p

Figure S3

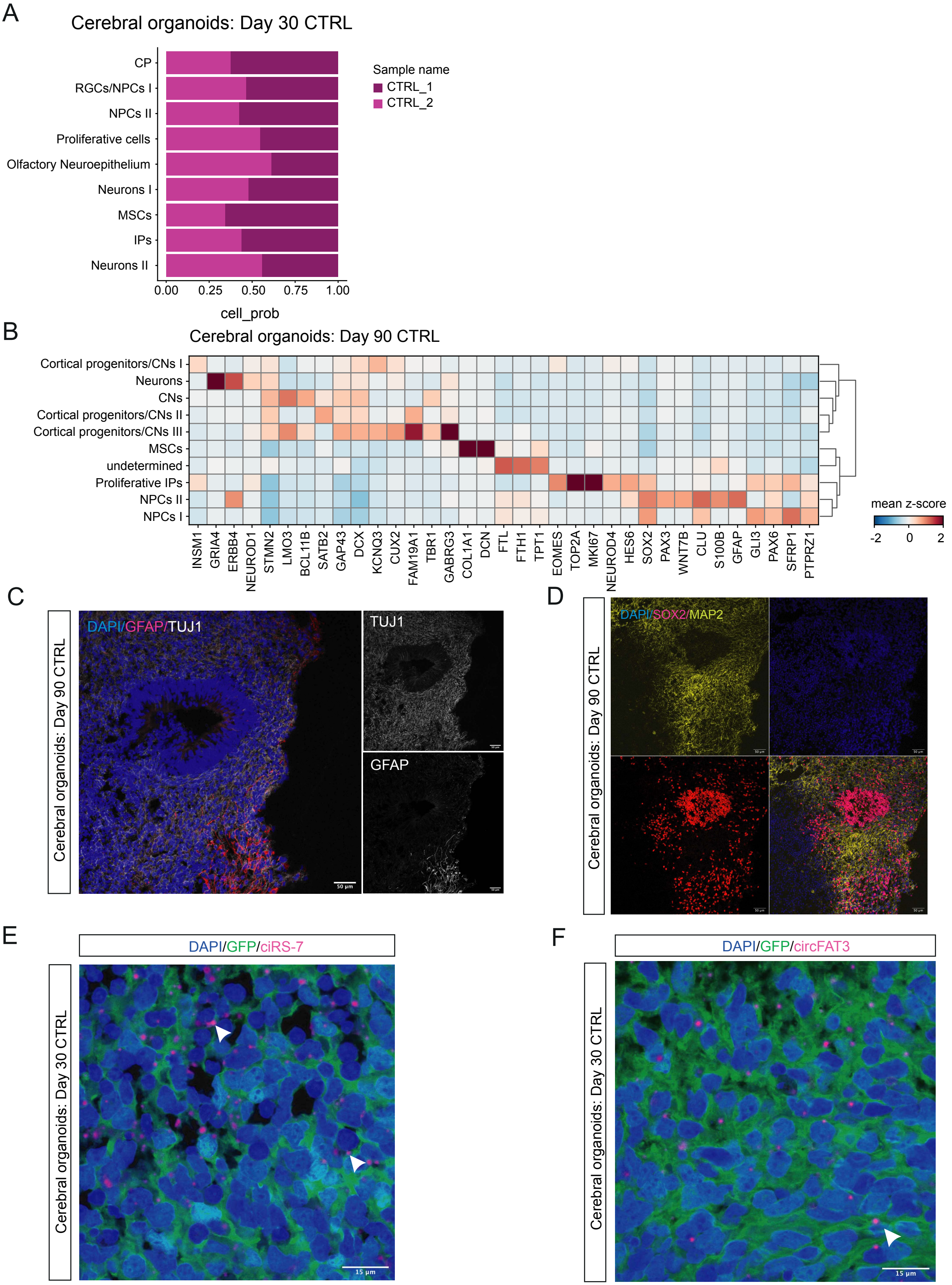
A
Cerebral organoids: Day 30 CTRL
CP
RGCs/NPCs I
NPCs II
Proliferative cells
Olfactory Neuroepithelium
Neurons I
MSCs
IPs
Neurons II
0.00
0.25
0.50
0.75
1.00
cell_prob
Sample name
CTRL_1
CTRL_2
B
Cerebral organoids: Day 90 CTRL
Cortical progenitors/CNs I
Neurons
CNs
Cortical progenitors/CNs II
Cortical progenitors/CNs III
MSCs
undetermined
Proliferative IPs
NPCs II
NPCs I
INSM1
GRIA4
ERBB4
NEUROD1
STMN2
LMO3
BCL11B
SATB2
GAP43
DCX
KCNQ3
CUX2
FAM19A1
TBR1
GABRG3
COL1A1
DCN
FTL
FTH1
TPT1
EOMES
TOP2A
MKI67
NEUROD4
HES6
SOX2
PAX3
WNT7B
CLU
S100B
GFAP
GLI3
PAX6
SFRP1
PTPRZ1
mean z-score
-2
0
2
C
Cerebral organoids: Day 90 CTRL
DAPI/GFAP/TUJ1
TUJ1
GFAP
D
Cerebral organoids: Day 90 CTRL
DAPI/SOX2/MAP2
E
DAPI/GFP/ciRS-7
Cerebral organoids: Day 30 CTRL
15 µm
F
DAPI/GFP/circFAT3
Cerebral organoids: Day 30 CTRL
15 µm

Figure S4

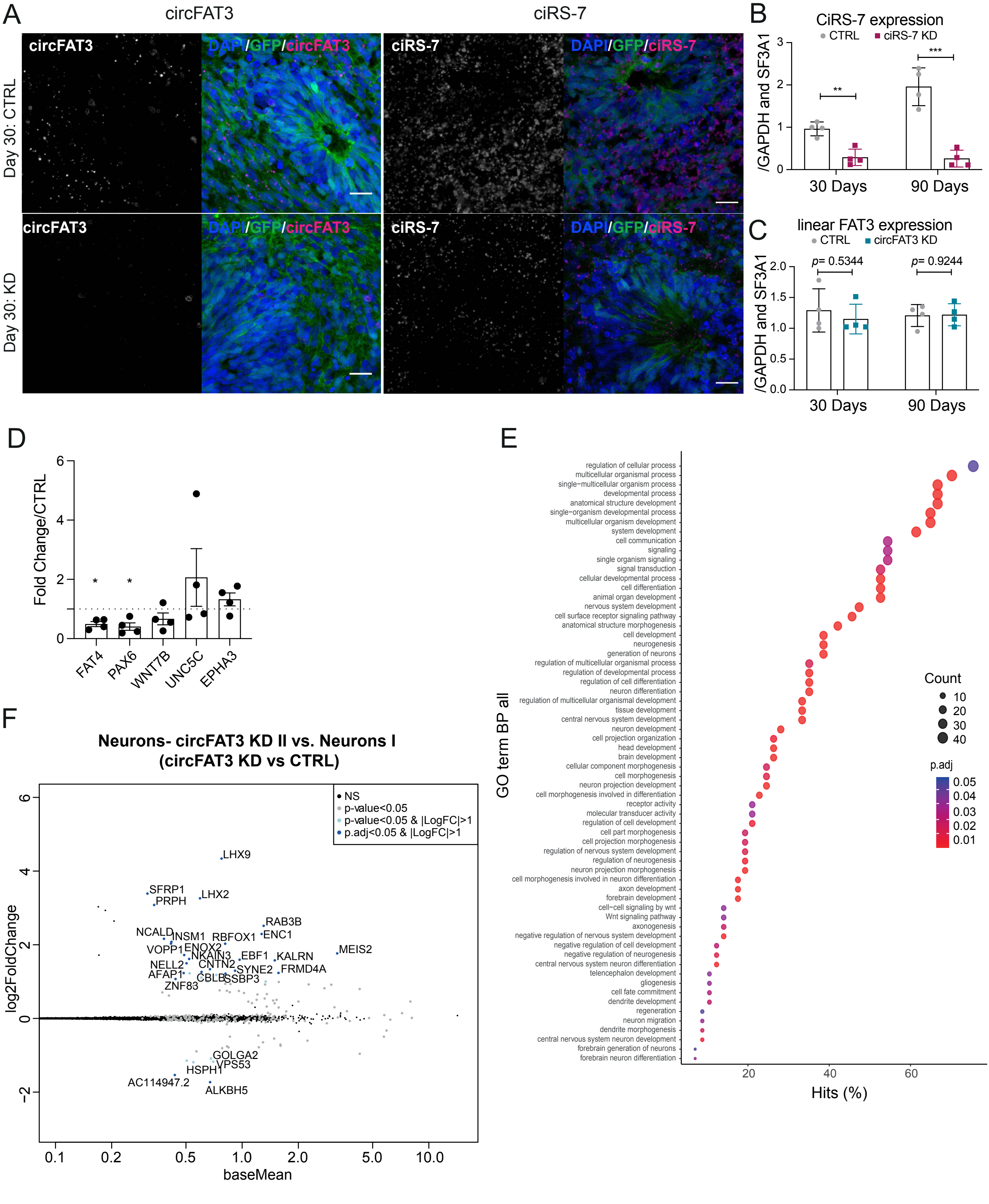
A
circFAT3
ciRS-7
Day 30: CTRL
Day 30: KD
DAPI/GFP/circFAT3
DAPI/GFP/ciRS-7
B
CiRS-7 expression
CTRL
ciRS-7 KD
/GAPDH and SF3A1
30 Days
90 Days
C
linear FAT3 expression
circFAT3 KD
p= 0.5344
p= 0.9244
D
Fold Change/CTRL
FAT4
PAX6
WNT7B
UNC5C
EPHA3
E
GO term BP all
Hits (%)
Count
p.adj
F
Neurons- circFAT3 KD II vs. Neurons I
(circFAT3 KD vs CTRL)
log2FoldChange
baseMean
NS
p-value<0.05
p-value<0.05 & |LogFC|>1
p.adj<0.05 & |LogFC|>1

Figure S5

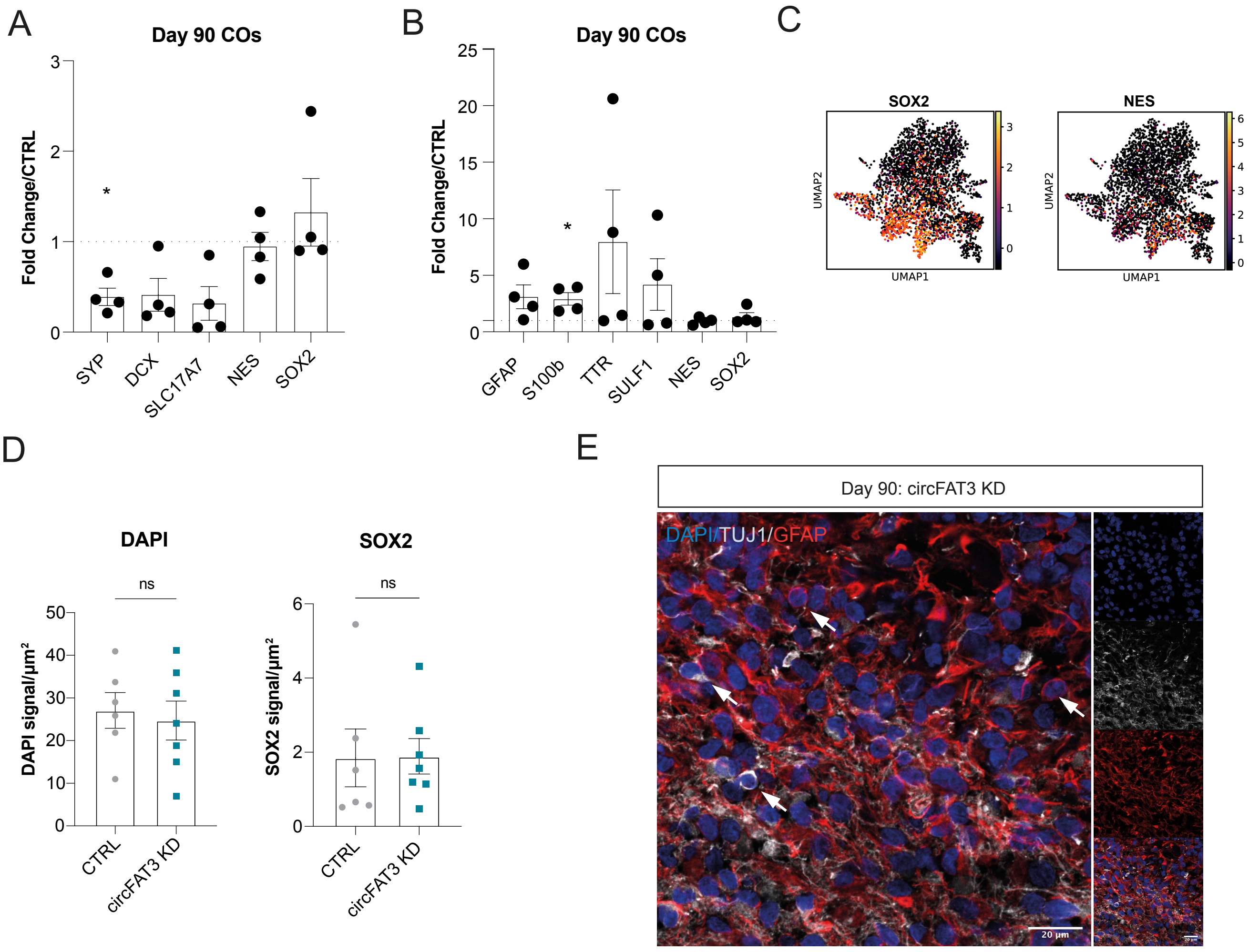

A
Day 90 COs
Fold Change/CTRL
SYP
DCX
SLC17A7
NES
SOX2
B
Day 90 COs
Fold Change/CTRL
GFAP
S100b
TTR
SULF1
NES
SOX2
C
SOX2
NES
UMAP1
UMAP2
D
DAPI
ns
DAPI signal/µm²
CTRL
circFAT3 KD
SOX2
ns
SOX2 signal/µm²
CTRL
circFAT3 KD
E
Day 90: circFAT3 KD
DAPI/TUJ1/GFAP
20 µm

Figure S6

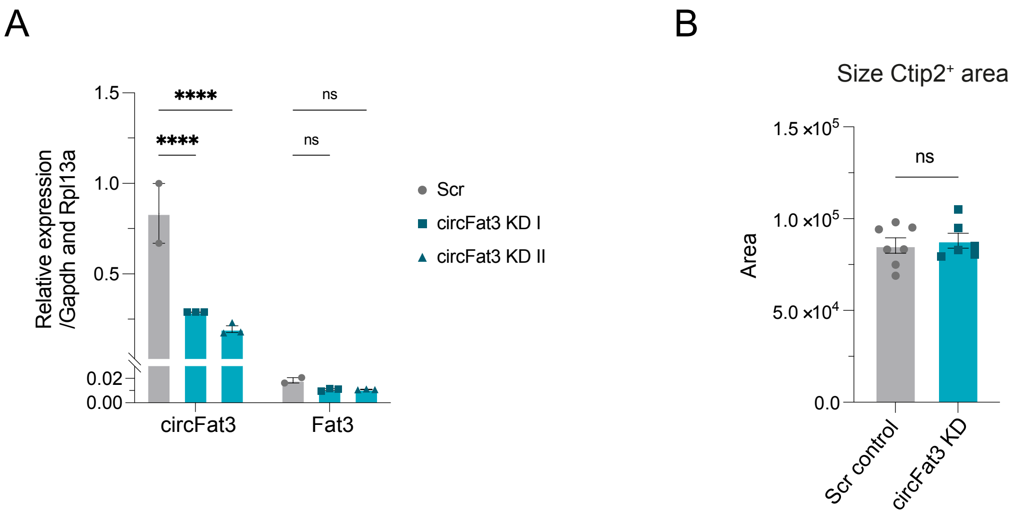
A
1.5
1.0
0.5
0.02
0.00
Relative expression
/Gapdh and Rpl13a
****
****
ns
ns
circFat3
Fat3
Scr
circFat3 KD I
circFat3 KD II
B
Size Ctip2⁺ area
1.5 ×10⁵
1.0 ×10⁵
5.0 ×10⁴
0.0
Area
ns
Scr control
circFat3 KD

Figure S7
